# Proteome-wide identification of lysine 2-hydroxyisobutyrylation reveals conserved and novel histone modifications in ***Physcomitrella patens***

**DOI:** 10.1038/s41598-017-15854-z

**Published:** 2017-11-14

**Authors:** Zhiming Yu, Jun Ni, Wei Sheng, Zhikun Wang, Yuhuan Wu

**Affiliations:** 1 College of Life and Environmental Sciences, Hangzhou Normal University, Hangzhou, 310018 China; 2 Department of Molecular Cellular and Developmental Biology, College of Letters & Science, University of California, Santa Barbara, CA 93116 USA

## Abstract

Protein lysine 2-hydroxyisobutyrylation (K_hib_) is a newly identified post-translational modification found in animal and yeast cells. Previous research suggested that histone K_hib_ is involved in male cell differentiation and plays a critical role in the regulation of chromatin functions in animals. However, information regarding protein K_hib_ in plants is still limited. In this study, using a specific antibody and LC-MS/MS methods, we identified 11,976 K_hib_ sites in 3,001 proteins in *Physcomitrella patens*. The bioinformatics analysis indicated that these K_hib_-modified proteins were involved in a wide range of molecular functions and cellular processes, and showed diverse subcellular localizations. Furthermore, an comparism of K_hib_ sites in histone proteins among human, mouse and *P*. *patens* found conserved sites in the H3 and H4 histone proteins and novel sites in H1, H2A and H2B histone proteins in *P*. *patens*. This is the first report on K_hib_ post-translational modifications in plants, and the study provides a comprehensive profile of K_hib_ sites in histone and non-histone proteins in *Physcomitrella patens*.

## Introduction

Protein post-translational modifications (PTMs) are covalent processing events that change a protein’s properties through proteolytic cleavage or the addition of a modifying group to one or more amino acids^1^. PTMs greatly exceed the number of proteins predicted by DNA coding capacities^2^. Presently, more than 200 different types of PTMs have been found, and these modifications dynamically regulate various biological events, such as subcellular localization, protein degradation, protein-protein interaction, conformational change, signal transduction and gene transcription^3–5^.

Among the PTMs, modifications to histone proteins are the most noticeable and attract the most attention. Although histone modifications do not change the DNA sequence, they are heritable and can be classified as epigenetic markers^6^. These histone modifications are proposed to store the epigenetic memories in the form of a “histone code” that regulates chromatin structure and gene activity^7^. In the last decade, with the development of high-specificity antibodies and high-resolution MS techniques, a number of novel sites and types of modifications have been found in histone proteins, which greatly expanded our knowledge of the histone code^8,9^.

Lysine 2-hydroxyisobutyrylation (K_hib_) is a newly identified modification first found in human and mouse histone proteins^10^. A western blotting analysis revealed that the K_hib_ modification to histones is evolutionarily conserved, existing also in *Drosophila* and yeast cells. Using chromatin immunoprecipitation sequencing, gene expression analyses and immunodetection, histone K_hib_ has been reported to associate with gene transcription in meiotic and post-meiotic male germ cells^10^. Recently, histone K_hib_ was also identified in trypanosomatids, indicating a wide distribution of histone K_hib_ in different species^11^. In spite of the important functions found in animal cells, information on K_hib_ in plant cells is still limited.

Bryophytes were the first land plants, originating between ~480 and 360 million years ago, and play an important part in the evolution of the plant kingdom^12^. Although tremendous morphological diversity exists between bryophytes and higher plants, studies indicate that many gene families controlling different morphologies in higher plants were already present in these earliest land plants^13^. Thus, bryophytes are powerful experimental tools for the elucidation of complex biological processes in plants^14^. Since the first isolation and characterization of mutants in the moss *Physcomitrella patens* ^15^, this plant has been used as a model system for physiological and developmental studies in plants^16–18^.

Here, using a high-specificity K_hib_ antibody and high-resolution MS techniques, we identified 11,976 K_hib_ sites in 3,001 proteins in *P*. *patens*. Additionally, a detailed analysis of histone K_hib_ revealed conserved and novel histone modificationsin *P*. *patens*. Our research on the nonhistone and histone K_hib_ modifications in *P*. *patens* will facilitate our understanding of the diverse and novel functions of K_hib_ in plant cells.

## Results and Discussion

### Detection and proteome-wide identification of Khib in *P. patens*

**Figure 1:**
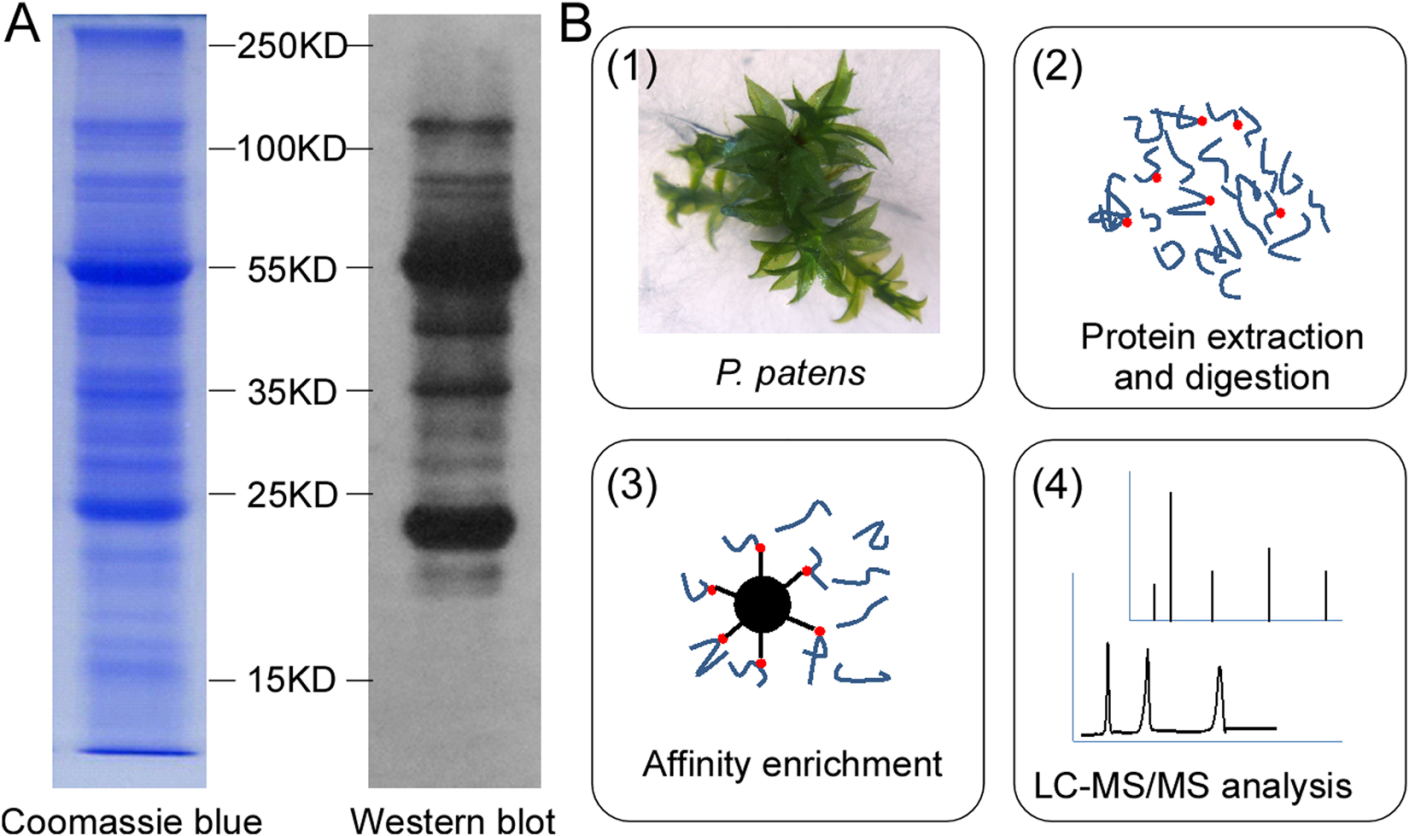
Workflow for large-scale detection of K_his_ modification sites in *P*. *patens*. (**A**) Western blotting analysis of total protein extracts to investigate the existence of K_hib_-modified proteins in *P*. *patens*. (**B**) The experimental strategy to identify K_hib_ modification sites in *P*. *patens*. Total protein extracted from *P*. *patens* was digested by trypsin. Peptides containing K_hib_ modification sites were enriched by immunoprecipitation with a K_hib_-specific antibody and subsequently analyzed by LC-MS/MS.

To investigate the presence of K_hib_ modifications in moss, western blotting was carried out in the total protein extracts of *P*. *patens*. As a result, a large number of protein bands, which occupy a wide protein mass range, were observed (Fig. 1A), demonstrating that K_his_ is highly abundant in moss.

To unambiguously identify K_hib_ sites in *P*. *patens*, peptides bearing this modification were analyzed by LC-MS/MS. Briefly, proteins isolated from a mix of plants grown separately were digested by trypsin. Then, the peptides bearing K_hib_ sites were enriched by affinity purification using K_hib_-specific antibodies. Subsequently, the enriched peptides were characterized by LC-MS/MS (Fig. 1B).

**Figure 2:**
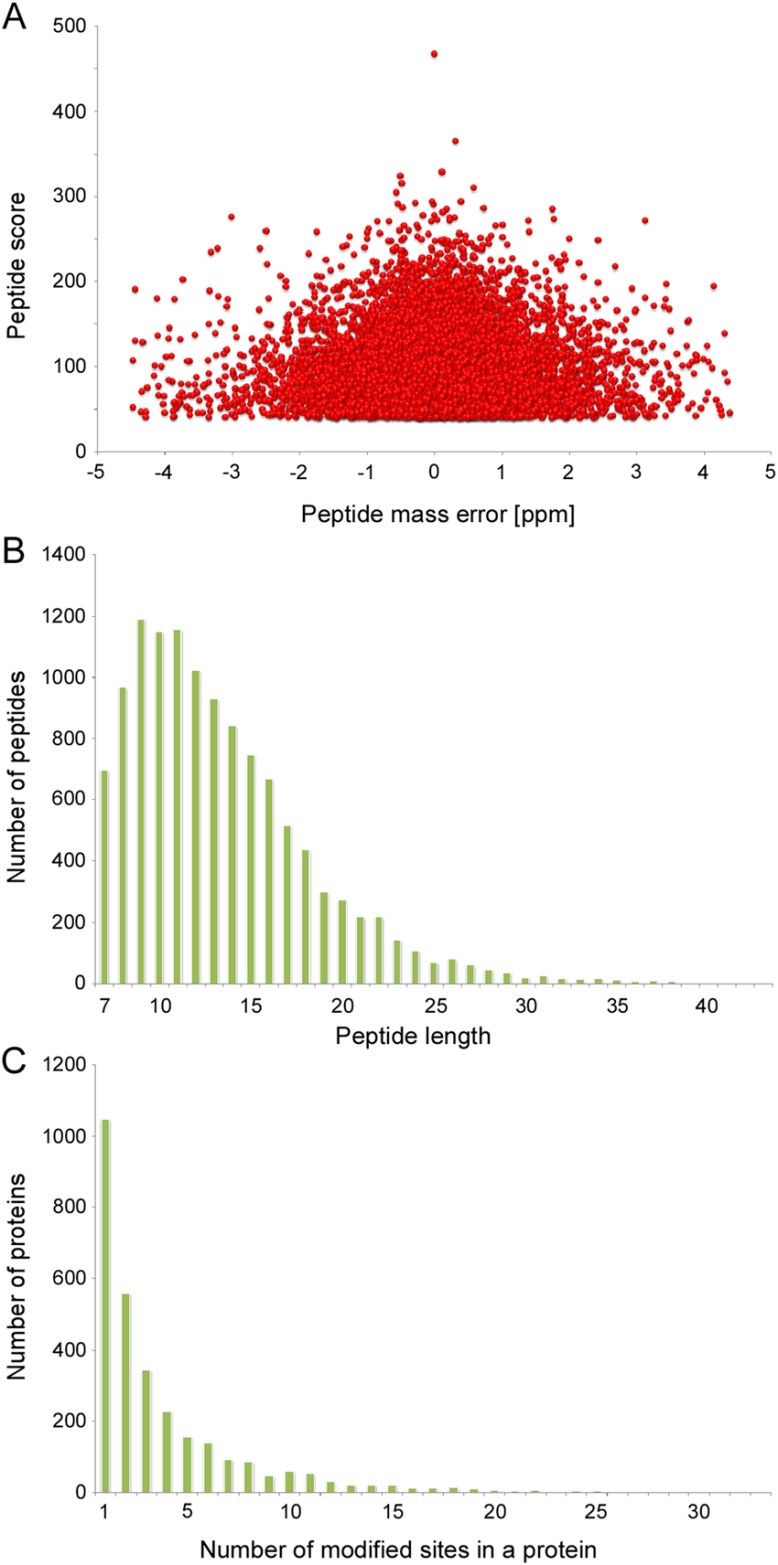
Quality control validation of MS data. (**A**) Distribution of the mass errors of the K_hib_-containing peptides. (**B**) Peptide length distribution. (**C**) Number of modified sites in a protein.

Of all the 13,138 peptides acquired, 11,976 peptides (11,976/13,138, 91.2%) in 3,001 proteins were identified with K_hib_ modifications (Table S1 and Table S2). To validate the quality of our MS data, the mass errors for all the K_hib_-containing peptides were checked. Most of the mass errors were less than 3 ppm (11,779/11,976, 98.4%), and 8,891 peptides (8,891/11,976, 74.2%) fell in a range of 1 ppm. This indicated a high degree of accuracy for the MS data (Fig. 2A and Table S3). The lengths of most identified peptides varied between 7 and 20 aa, with a peak length of 9 aa for nearly 1,200 peptides (10.0%) (Fig. 2B). The lengths of these peptides were similar to previous research, and this was in agreement with the property of tryptic peptides^19,20^. In addition, the distribution of modified sites within individual proteins was determined, and more than 1,000 proteins contained only 1 site and a very low percentage contained more than 10 sites (Fig. 2C).

### Characterization of Khib-modified proteins in *P. patens*

**Figure 3:**
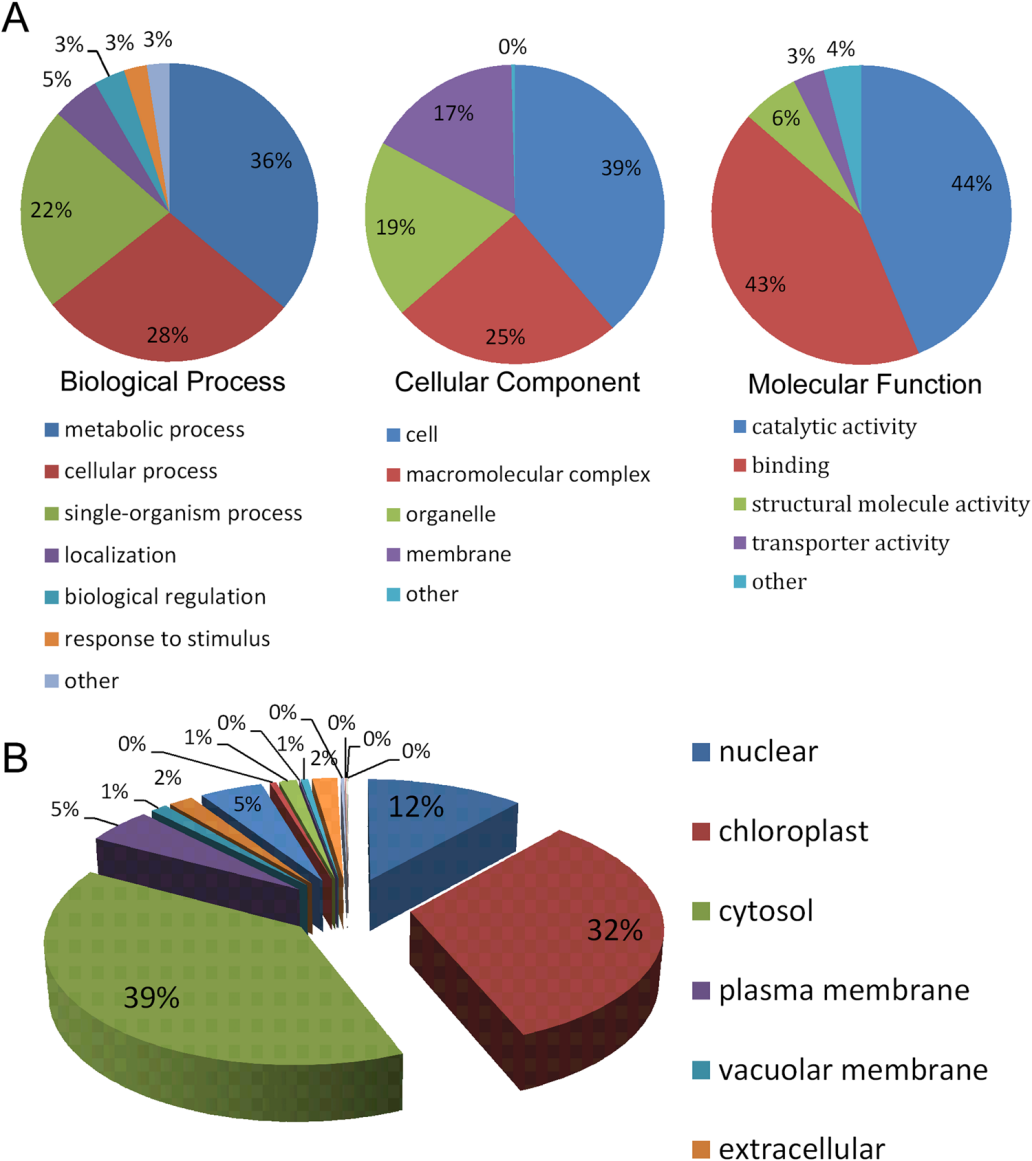
Analysis of K_hib_-modified proteins. (**A**) Functional classification of K_hib_-modified proteins. (**B**) Subcellular localizations of K_hib_-modified proteins in *P*. *patens*.

Gene ontology (GO) classifications are frequently used to determine possible protein functions and localizations^21^. Here, the GO-term classification was performed by searching the Uniprot-GOA database. The K_hib_-modified proteins were involved in a diverse range of biological processes, cellular components and molecular functions. In the GO terms of ‘biological process’, most of the modified proteins were classified into ‘metabolic process’ (36%), ‘cellular process’ (28%) and ‘single-organism process’ (22%). In the GO terms of ‘cellular component’, most of the modified proteins were distributed in ‘cell’ (39%), ‘macromolecular complex’ (25%), ‘organelle’ (19%) and ‘membrane’ (17%). In the GO terms of ‘molecular function’, most of the modified proteins were distributed in two categories, ‘catalytic activity’ (44%) and ‘binding’ (43%) (Fig. 3A). This is the first attempt to classify K_hib_-modified proteins, thus we compared our result to the classification of other protein modifications. Protein succinylation is another kind of modification, recent analysis revealed that the GO classification of succinylated proteins is very similar with K_hib_-modified proteins, with the largest classes ‘metabolic process’ in ‘biological process’, and ‘catalytic activity’ in ‘molecular function’^19,20^. This indicated that various PTMs may be needed to facilitate these processes and activities.

The subcellular localizations of modified proteins were also analyzed in *P*. *patens*. In total, 39% of the proteins were estimated to localize in the cytosol, 32% in chloroplasts and 12% in nuclei (Fig. 3B and Table S3). Recent research on lysine succinylation-modified proteins in *Taxus*, a hybrid Taxus species containing a high content of taxol, also shows a preference for subcellular localizations in cytosol, chloroplasts and nuclei^19^. In animal cells, protein K_hib_ has only been analyzed in nuclear-localized histone proteins^10^. The diversity of K_hib_-modified proteins’ subcellular localizations indicates that, besides from histone proteins in the nucleus, K_hib_ may regulate functions of other proteins in different organelles.

### Enrichment analysis of Khib-modified proteins in *P. patens*

To investigate the preferred protein types, metabolic pathways and protein domains of K_hib_-modified proteins, the GO, KEGG and domain enrichments of K_hib_ proteins in *P*. *patens* were evaluated. In the GO analysis, the terms of ‘oxidoreductase activity, acting on CH-OH group of donors’ (GO: 0016614), ‘structural molecule activity’ (GO: 0005198), ‘cofactor binding’ (GO: 0048037), ‘coenzyme binding’ (GO: 0050662) and ‘ligase activity’ (GO: 0016874) were most significantly enriched in ‘molecular function’. In the ‘cellular component’, the K_hib_-modified proteins were enriched in ‘ribosome’ (GO: 0005840), ‘ribonucleoprotein complex’ (GO: 0030529) and ‘cytoplasmic part’ (GO: 0044444). In the ‘biological process’, the terms of ‘oxoacid metabolic process’ (GO: 0043436), ‘organonitrogen compound biosynthetic process’ (GO: 1901566), ‘small molecule metabolic process’ (GO: 0044281), ‘alpha-amino acid metabolic process’ (GO: 1901605), ‘organonitrogen compound metabolic process’ (GO: 1901564), ‘translation’ (GO: 0006412) and ‘organic acid metabolic process’ (GO: 0006082) were the most enriched (Table S4).

For the KEGG enrichment analysis, 25 significantly enriched pathways were identified. The top three highest enriched metabolic pathways were ‘microbial metabolism in diverse environments’ (KO 01120), ‘carbon metabolism’ (KO 01200) and ‘biosynthesis of antibiotics’ (KO 01130) (Table S5).

In addition, the domain enrichment analysis of K_hib_-modified proteins showed that 25 domains were significantly enriched, with the’thioredoxin-like fold’ domain being the most significant (Table S6).

Compared with succinylated proteins, K_hib_-modified proteins showed different enrichment patterns^19,20^. Considering the similarity of GO classification between succinylated and K_hib_-modified proteins, we propose that although numerous PTMs are needed in various processes and activities, they play different roles and enriched in specific pathways. Until now, only a small fraction of PTMs have been functionally characterized^4^. Thus, our results may facilitate the functional characterization of K_hib_-modified proteins in future.

### Motif analysis in identified Khib-modified peptides

**Figure 4:**
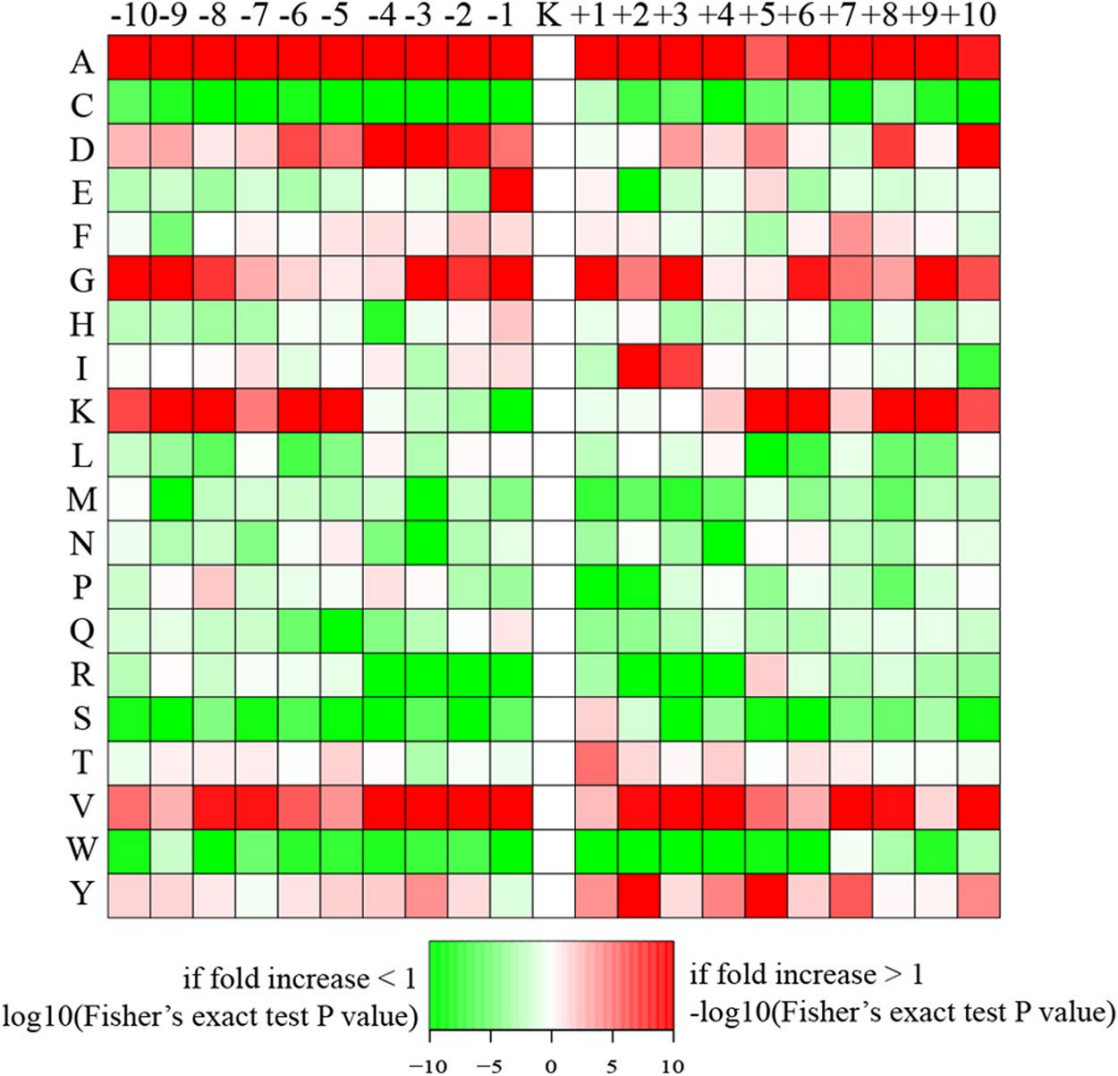
Sequence analyses of amino acids flanking the K_hib_ sites in *P*. *patens*. Heatmap shows enrichment (red) and depletion (green) of amino acids in specific positions flanking K_hib_ in moss.

To evaluate the motif patterns in K_hib_-modified peptides, we used Motif-X to extract overrepresented and underrepresented patterns in the identified K_hib_-modified peptides. A heatmap analysis of enriched and depleted amino acids showed that some were specifically enriched the near the K_hib_ sites. Alanine residues were overrepresented in almost all of the positions in the K_hib_ sites, and aspartic acid, glycine and valine residues were overrepresented in the majority of positions in the K_hib_ sites. Interestingly, lysine residues were overrepresented in the −10–−5 and +5– +10 positions, leaving the nearest positions underrepresented. In addition, cysteine, serine and tryptophan residues were underrepresented in the majority of positions in the K_hib_ sites (Fig. 4). Interestingly, the K_hib_-modified motif pattern was different from the succinylation-modified motif in *Taxus*, but similar to the succinylation-modified motif in tomato^19,20^. This surprising result indicated that the mechanisms of different PTMs may be much more complex than originally thought.

### Both conserved and novel histone Khib sites in *P. patens*

**Figure 5:**
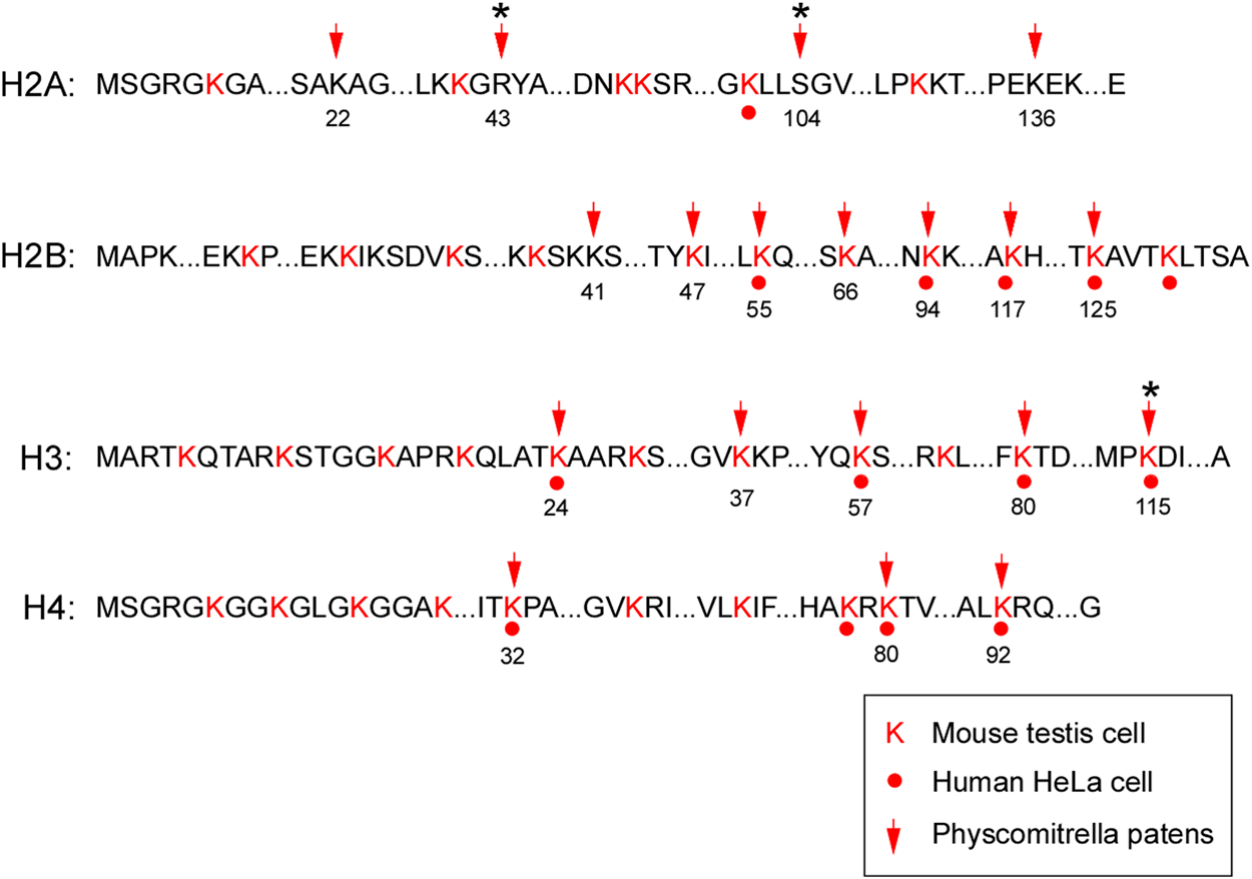
K_hib_ sites in human, mouse and *P*. *patens* histone proteins. The protein sequences of histones in human are shown, with the modified lysines marked in red. The positions of modified lysines in mouse and moss are marked with dots and arrows, respectively. The canonical histone sequences are shown as the base sequences. K_hib_ modifications in variant histones are marked by asterisks.

K_hib_ was first identified as a histone modification in human and mouse cells, and was regarded as a conserved modification in *Drosophila* and yeast cells^10^. Thus, we investigated whether this histone modification also occurred in moss. As a result, we identified 20 K_hib_ sites in histone proteins of *P*. *patens* (Figure S1). There was one site in histone H1, four sites in histone H2A, seven sites in histone H2B, five sites in histone H3, and three sites in histone H4 (Fig. 5 and Table S7).

Considering that most of the K_hib_ modified histone proteins are canonical histones, we carried out a homology analysis of canonical histone proteins among human, mouse and *P*. *patens*. Because of the extremely high histone protein homology between human and mouse, we only calculated the similarity of histone proteins between human and *P*. *patens*. The protein sequence alignment analysis showed that H3 and H4 histone proteins are the most conserved of the histone proteins with the similarity 97.1% and 98.1% respectively. Compared with H3 and H4 proteins in human, H3 contained four amino acid substitutions with two of them resulted in similar amino acid properties (lysine to arginine, and leucine to methionine) and H4 contained one amino acid substitution which resulted in similar amino acid properties (isoleucine to valine) in *P*. *patens*. Although sequence homology was observed for H2A and H2B between human and *P*. *patens*, deletions, insertions and a large number of substitutions were also observed, resulting in the lower homology of H2A and H2B histone proteins between the two organisms compared with the levels for H3 and H4 (the similarities are 74.4% and 67.8% respectively). In addition, the H1 histone protein in *P*. *patens* was very different from the human H1.2 protein, and they shared limited homology (the similarity is 28.7%) (Figure S2).

A detailed analysis revealed both conserved and novel K_hib_ sites in *P*. *patens*. For the most conserved histone protein, H4, all three K_hib_ sites in *P*. *patens* were observed in both mouse testis cells and human HeLa cells. For the histone protein H3, four K_hib_ sites in *P*. *patens* were observed in both mouse testis cells and human HeLa cells, while one K_hib_ site (H3K37) was only observed in mouse testis cells (Fig. 5 and Figure S3). Histone proteins H2A and H2B are not as conserved as H3 and H4 (Figure S2), and the histone K_hib_ pattern in *P*. *patens* was more complex. H2B contained the highest number of K_hib_ sites compared with other histone proteins in *P*. *patens*. Of the seven sites, four sites (H2BK55, H2BK94, H2BK117 and H2BK125) were observed in both mouse and human cells, while two sites (H2BK47 and H2BK66) were observed only in mouse cells. For one K_hib_ site (H2BK41), the modification was novel, neither identified in human nor mouse cells. All the K_hib_ sites detected for H2A and H1 in *P*. *patens* were not found in human or mouse cells (Fig. 5, Figure S3 and Table S7). Thus, K_hib_ sites were conserved in H3 and H4 histone proteins and changed dramatically in H1, H2A and H2B histone proteins in *P*. *patens*.

K_hib_ in a histone was first identified in mouse testis cells, and the modification was important for specific gene transcription events in meiotic and post-meiotic cells^10^. In our experiment, the number of histone K_hib_ modifications in *P*. *patens* were found between the germ and non-germ cells, and some K_hib_ sites (H2BK47, H2BK66 and H3K37) were observed in both moss and mouse germ cells but not human non-germ cells (Fig. 5). To investigate whether the germ cells were differentiated in *P*. *patens* ^22^, the plants were dissected under a microscope and no trace of an antherid or archegonium was found (Figure S4). Thus, the cells in our experiment were non-germ cells. Nevertheless, the post-meiotic cells in mouse testis are haploid gametophyte, which dominates the life cycle of *P*. *patens* ^16^. Thus, the extra K_hib_ sites in *P*. *patens*, compared with those of HeLa cells in human, which are diploid sporophyte, may be involved in the maintenance of the haploid gametophyte status. Thus, it will be interesting to investigate the K_hib_-modified sites of histone proteins in germ cells containing antherid or archegonium in the future.

## Conclusions

In summary, we presented a large-scale proteome-wide identification of K_hib_ in *P*. *patens*, an important model moss plant. The K_hib_-modified proteins were estimated to be distributed in various cellular compartments and involved in a broad spectrum of processes. Moreover, an analysis of K_hib_ sites in histone proteins revealed conserved sites in H3 and H4 histone proteins and novel sites in H1, H2A and H2B histone proteins. This is the first report on the K_hib_ modification in plants and our results provide novel insights into the functions of K_hib_ proteins.

## Materials and Methods

### Plant materials and protein extraction

Haploid gametophyte stage of *P*. *patens* was transplanted and cultured on solid Knop medium plate according to the methods mentioned previously^23^. The moss was cultured in growth chamber at 25 °C under day time and 22 °C under night time. The photoperiods were 16 h light/8 h dark cycles. After 4 weeks, at least 20 g of fresh moss grown separately was yielded and the morphology of *P*. *patens* was evaluated by optical-stereo microscopy (Nikon SMZ 1500 stereo microscope, Tokyo, Japan).

For protein extraction, plant sample was submerged in lysis buffer (8 M urea, 2 mM EDTA, 3 μM TSA, 50 mM NAM, 10 mM DTT and 1% protease Inhibitor Cocktail) and sonicated for three times on ice using a high intensity ultrasonic processor (Scientz). After centrifugation at 2000g at 4 °C for 10 min, the protein in the supernatant was precipitated with 15% TCA for 2 h at −20 °C. The supernatant was discarded after centrifugation at 2000 g at 4 °C for 10 min, and the remaining precipitate was washed with cold acetone for at least three times. For the analysis of protein, it was redissolved in buffer (8 M urea, 100 mM NH_4_CO_3_, pH 8.0) and the 2-D Quant kit (GE Healthcare) was used to determine the protein concentration.

### Trypsin digestion, HPLC fractionation and affinity enrichment of protein

For the trypsin digestion, the protein solution was treated at room temperature in darkness with 10 mM dithiothreitol (DTT, Sigma) for 1 h at 37 °C and 20 mM iodoacetamide (IAA, Sigma) for 45 min. To eliminate the effects of urea in trypsin digestion, 100 mM NH_4_CO_3_ was used to dilute the protein sample. The final concentration of urea should be less than 2 M. The trypsin (Promega) was added to the protein solution at the mass protease:protein ratio of 1:50 for the first overnight-digestion and 1:100 for the second 4h-digestion.

The peptides were fractionated offline by high pH reversed-phase HPLC using Agilent 300Extend C18 column (5 μm particles, 4.6 mm ID, 250 mm length). First, peptides were separated into 80 fractions with a gradient of 2% to 60% acetonitrile in 10 mM ammonium bicarbonate pH 10 over 80 min. Then, the peptides were combined into 6 fractions equally and dried by vacuum centrifuging.

In order to enrich the K (2-OHib) peptides, tryptic peptides were dissolved in NETN buffer (100 mM NaCl, 1 mM EDTA, 50 mM Tris-HCl, 0.5% NP-40, pH 8.0), and then incubated with pre-washed antibody conjugated with beads (PTM Biolabs, PTM-801) at 4 °C overnight with gentle shaking. After incubation, the beads were washed with NETN buffer for four times and ddH2O for two times. Then, the peptides were eluted from the beads with 0.1% TFA. The eluted peptides were combined and vacuum-dried. The resulting peptides were cleaned with C18 ZipTips (Millipore) before LC-MS/MS analysis.

### Quantitative Proteomic Analysis by LC-MS/MS

The enriched peptides were dissolved in 0.1% formic acid (Fluka), and loaded onto a reversed-phase pre-column (Acclaim PepMap 100, Thermo Scientific, 164568, 150 mm) directly. A reversed phase analytical column (Acclaim PepMap RSLC, Thermo Scientific, 164534, 150 mm) was used to separate the peptide. The constant flow of 300 nl/min was provided by an EASY-nLC 1000 UPLC system. The gradient was started with an increase from 6% to 22% solvent B (0.1% formic acid in 98% acetonitrile (Fisher Chemical)) for 24 min, then 22% to 40% for 8 min and climbing to 80% in 5 min, then holding at 80% for the last 3 min. The resulting peptides were analyzed by Q Exactive^TM^ Plus hybrid quadrupole-Orbitrap mass spectrometer (ThermoFisher Scientific).

The peptides were subjected to NSI source followed by tandem mass spectrometry (MS/MS) in Q Exactive^TM^ plus (Thermo) coupled online with the UPLC. Intact peptides were detected in the Orbitrap at a resolution of 70000. Peptides were selected for MS/MS using NCE setting as 30; ion fragments were detected in the Orbitrap at a resolution of 17500. A data-dependent procedure that alternated between one MS scan followed by 20 MS/MS scans was applied for the top 20 precursor ions above a threshold ion count of 5E3 in the MS survey scan with 15.0 s dynamic exclusion. The electrospray voltage applied was 2.0 kV. Automatic gain control (AGC) was used to prevent overfilling of the orbitrap; 5E4 ions were accumulated for generation of MS/MS spectra. For MS scans, the m/z scan range was 350 to 1800. Fixed first mass was set as 100 m/z.

MaxQuant integrated with Andromeda search engine (v.1.5.1.8) was used to process the resulting MS/MS data. The data was searched against the database of *uniprot_physcomitrella* concatenated with reverse decoy database. Trypsin/P was specified as cleavage enzyme allowing up to 5 modifications, 4 missed cleavages per peptide and max. 5 charges. Mass error was set to 0.02 Da for fragment ions and 10 ppm for precursor ions. Acetylation on protein N-terminal, 2-hydroxyisobutyrylation on Lys and oxidation on Met were specified as variable modifications, and carbamidomethylation on Cys was specified as fixed modification. The thresholds of false discovery rate (FDR) for peptide, protein and modification site were specified at 1%. The minimum peptide length was set at 7. All the other parameters in MaxQuant were set to default values. The site localization probability was set as>0.75.

### Enrichment of Gene Ontology analysis

Gene Ontology (GO) annotation of proteome was derived from the UniProt-GOA database (www. http://www.ebi.ac.uk/GOA/). The identified protein ID was first converted to UniProt ID, then mapped to GO IDs by protein ID. When an identified protein was not annotated by UniProt-GOA database, the InterProScan software was used to annotated protein’s GO functional based on the alignment of protein sequence. For functional enrichment, proteins were classified into three categories: biological process, cellular compartment and molecular function. In each category, the two-tailed Fisher’s exact test was used to test the enrichment of identified proteins against all database proteins. Correction for multiple hypothesis testing was carried out using standard false discovery rate control methods. The GO with a corrected p-value < 0.05 is considered significant.

### Motif and protein domain analysis

Motif-x was used to analyze the model of motif sequences constituted by amino acids in specific positions of modified-21-mers (10 amino acids upstream and downstream of the site) in all protein sequences. All the protein sequence databases were used as background database parameter, with other parameters default setting. Domains of identified proteins were annotated by InterProScan, using the InterPro domain database, based on the alignment of protein sequences. A two-tailed Fisher’s exact test was employed to test the enrichment of the identified protein against all database proteins. Correction for multiple hypothesis testing was carried out using standard false discovery rate control methods and domains with a corrected p-value < 0.05 were considered significant.

### KEGG pathway analysis

For KEGG annotation, first, KEGG online service KAAS was used to annotated protein’s KEGG database description. Then, using KEGG online service tools KEGG mapper, the annotation results were mapped on the KEGG pathway database. For KEGG enrichment analysis, the KEGG database was used to identify protein against all database proteins. Correction for multiple hypothesis testing was carried out using standard false discovery rate control methods. The pathway with a corrected p-value < 0.05 was considered significant. These pathways were classified into hierarchical categories according to the KEGG website.

## Electronic supplementary material

Supplementary figures

Table S1

Table S2

Table S3

Table S4

Table S5

Table S6

Table S7
