# Supplementary material for: Proteome-wide identification of lysine 2-hydroxyisobutyrylation reveals conserved and novel histone modifications in Physcomitrella patens
Source: Sci Rep. 2017 Nov 14;7:15553. doi: 10.1038/s41598-017-15854-z (PMC5686104; doi:10.1038/s41598-017-15854-z)

# Proteome-wide identification of lysine 2-hydroxyisobutyrylation reveals conserved and novel histone modifications in *Physcomitrella patens*

Zhiming Yu[1, 3], Jun Ni[1, 3], Wei Sheng [1], Zhikun Wang[2], Yuhuan Wu[1 *]

[1]College of Life and Environmental Sciences, Hangzhou Normal University, Hangzhou 310018, China
[2] Department of Molecular Cellular and Developmental Biology, College of Letters & Science, University of California, Santa Barbara, CA, 93116
[3] These authors contributed equally to this work.

**Figure S1. MS/MS spectra for all histone $K_{hib}$ sites.**

**Figure S2. Canonical histone protein alignments among human, mouse and *P. patens*. (A)** H1.2, **(B)** H2A, **(C)** H2B, **(D)** H3 and **(E)** H4. Amino acid substitutions are indicated by red arrow heads.

**Figure S3. Protein alignments of different H2A and H3 proteins in *P. patens*.** $K_{hib}$ stites were marked with red dots.

**Figure S4. No differentiated antherid or archegonium are found in *P. patens*. (A)** The morphology of *P. patens* used in our experiment. **(B)** Microscopy of dissected moss plants. There is no differentiated antherid or archegonium. Bars = 0.5 mm.

**Table S1. Basic information on $K_{hib}$-modified proteins in *P. patens*.** Protein accession: protein accession number from the database used for the search. Position: position of the post-translational modification in the protein. Amino acid: amino acid modified by the post-translational modification. Protein description: protein function. Gene name: indicates the name of the gene that codes for the protein sequence. Score: analysis result of Mascot, a simple rule to be used to judge whether a result is significant or not. Modified sequence: modified peptide sequences with localization probabilities. Mass error (ppm): parts per million deviation of peptide mass errors between the theoretical and actual values. Charge: carried charge of the peptide. Localization probability: probability of a certain amino acid having this modification in this peptide.

**Table S2. Summary of LC-MS/MS results.**

**Table S3. Annotation and description of $K_{hib}$-modified proteins in *P. patens*.**

**Table S4. GO enrichment analysis of $K_{hib}$-modified proteins in *P. patens*.**

**Table S5. KEGG enrichment analysis of $K_{hib}$-modified proteins in *P. patens*.**

**Table S6. Protein domain enrichment analysis of $K_{hib}$-modified proteins in *P. patens*.**

**Table S7. $K_{hib}$-modified histone proteins in *P. patens*.** Information of canonical histones are marked in red.

# Figure S1

| Raw file | Scan | Method | Score | m/z |
|---|---|---|---|---|
| 3 | 2158 | FTMS; HCD | 110.12 | 472.78 |

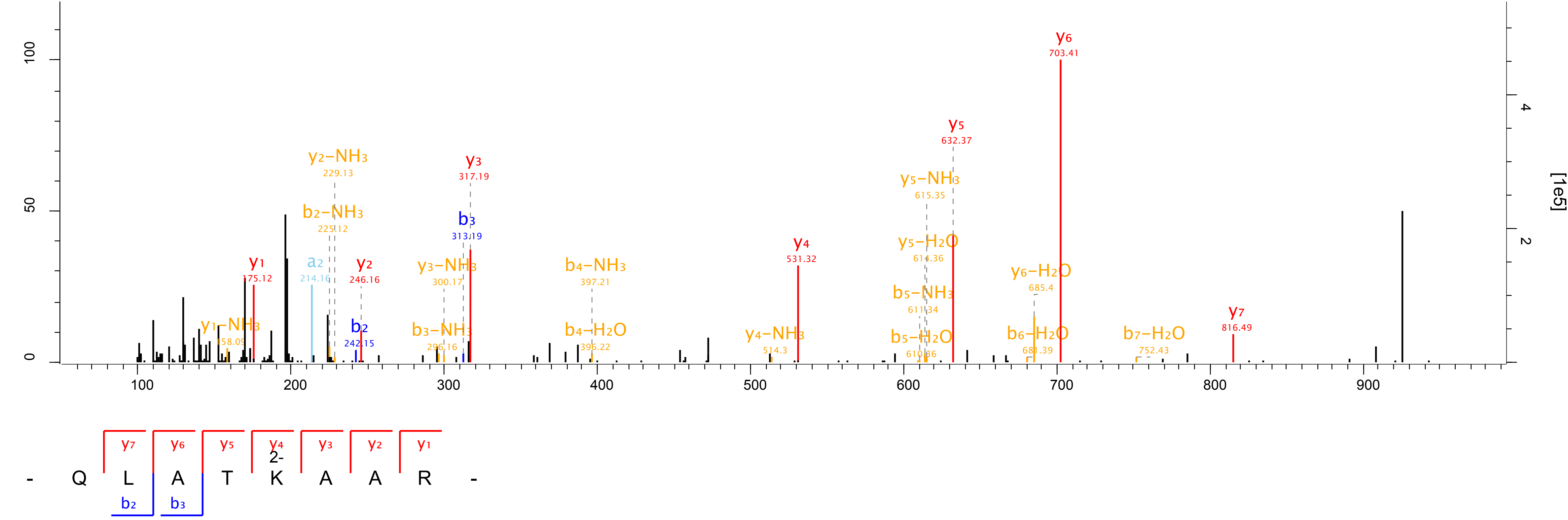

| Raw file | Scan | Method | Score | m/z |
|---|---|---|---|---|
| 2 | 1391 | FTMS; HCD | 87.72 | 711.9 |

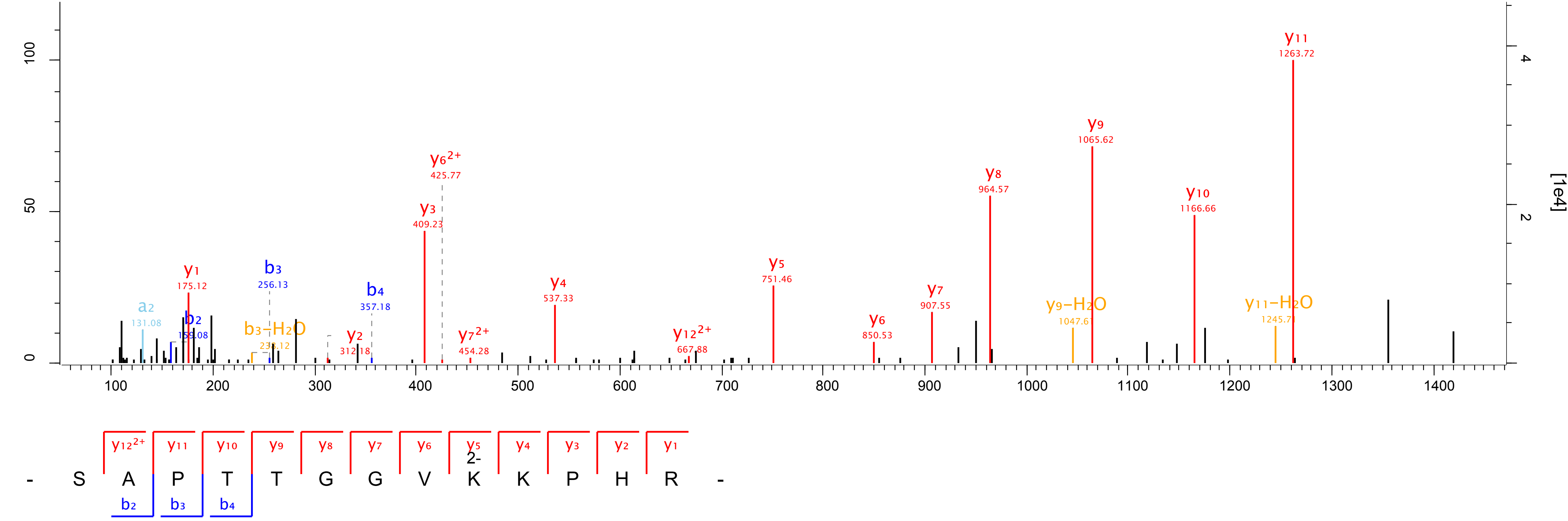

| Raw file | Scan | Method | Score | m/z |
|---|---|---|---|---|
| 6 | 5404 | FTMS; HCD | 146.16 | 732.93 |

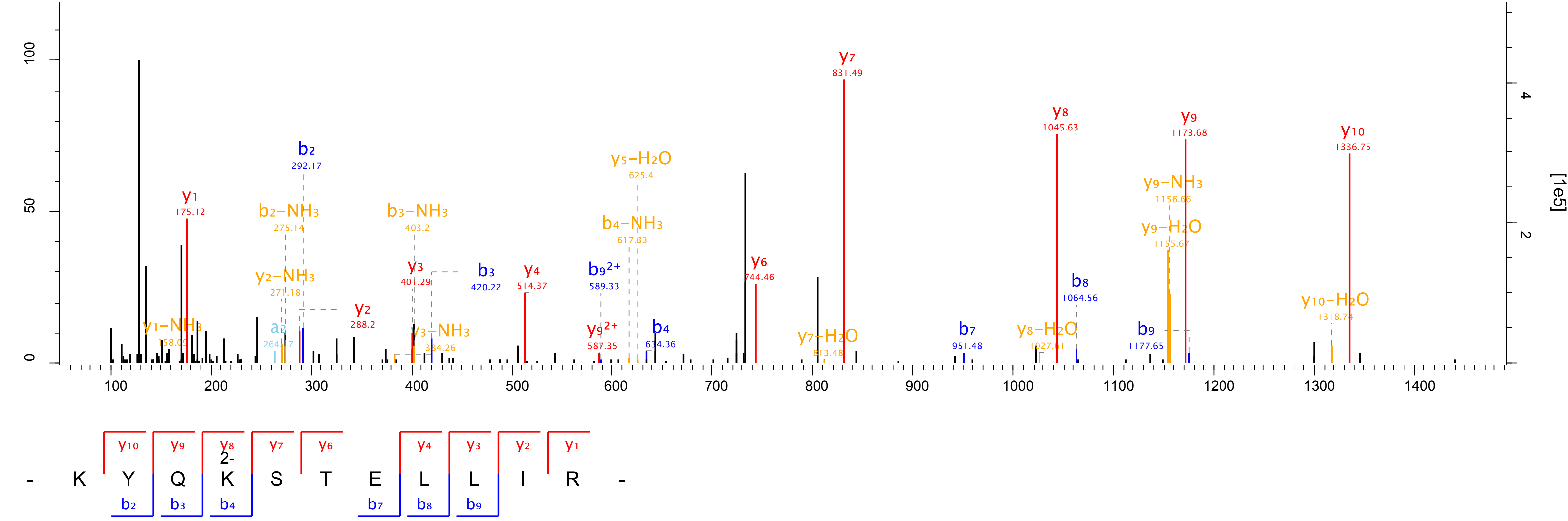

| Raw file | Scan | Method | Score | m/z |
| --- | --- | --- | --- | --- |
| 6 | 8986 | FTMS; HCD | 111.86 | 711.37 |

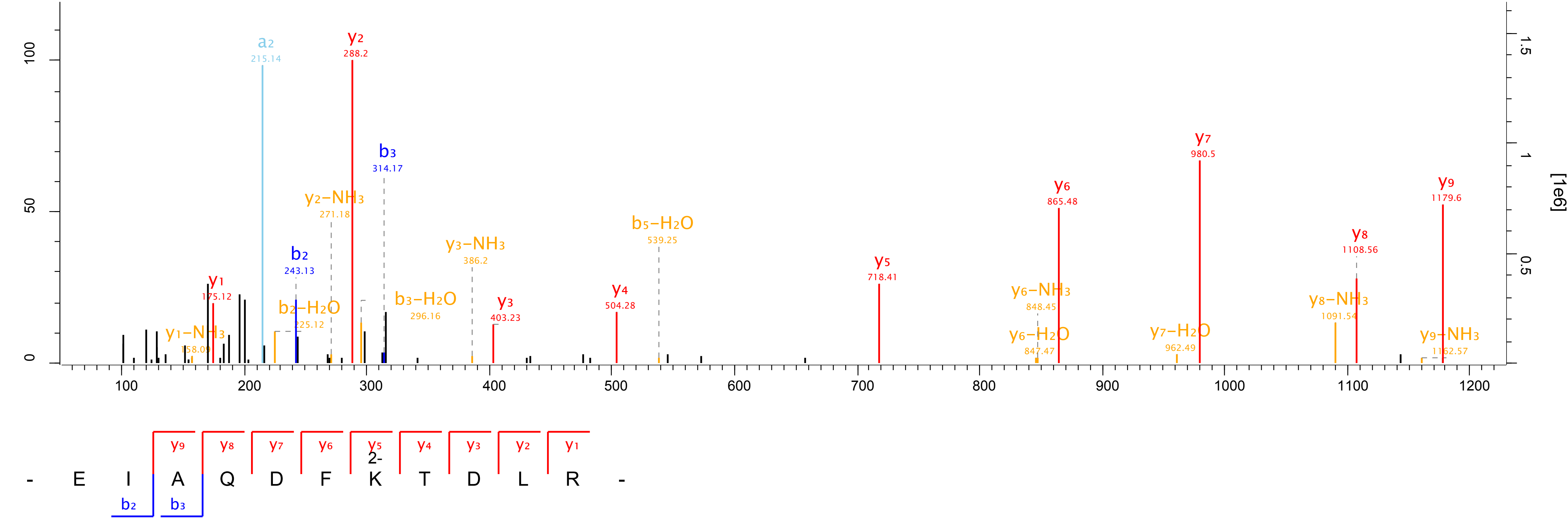

| Raw file | Scan | Method | Score | m/z |
|---|---|---|---|---|
| 2 | 7558 | FTMS; HCD | 65.84 | 633.83 |

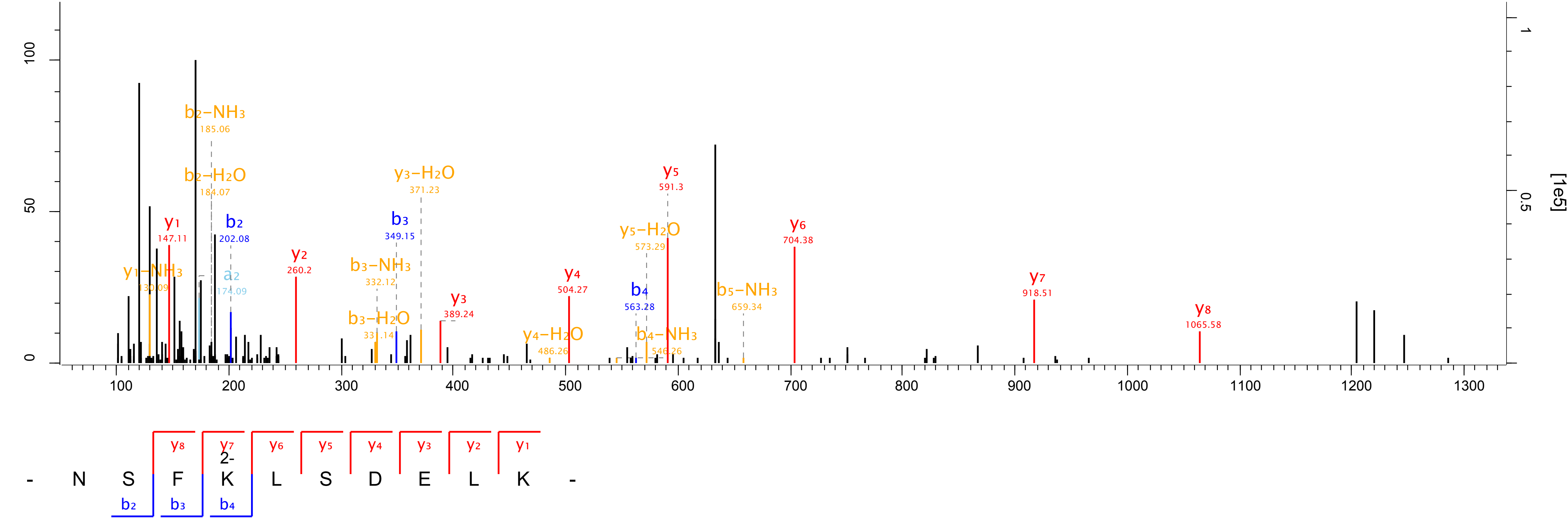

| Raw file | Scan | Method | Score | m/z |
|---|---|---|---|---|
| 4 | 1749 | FTMS; HCD | 60.51 | 440.73 |

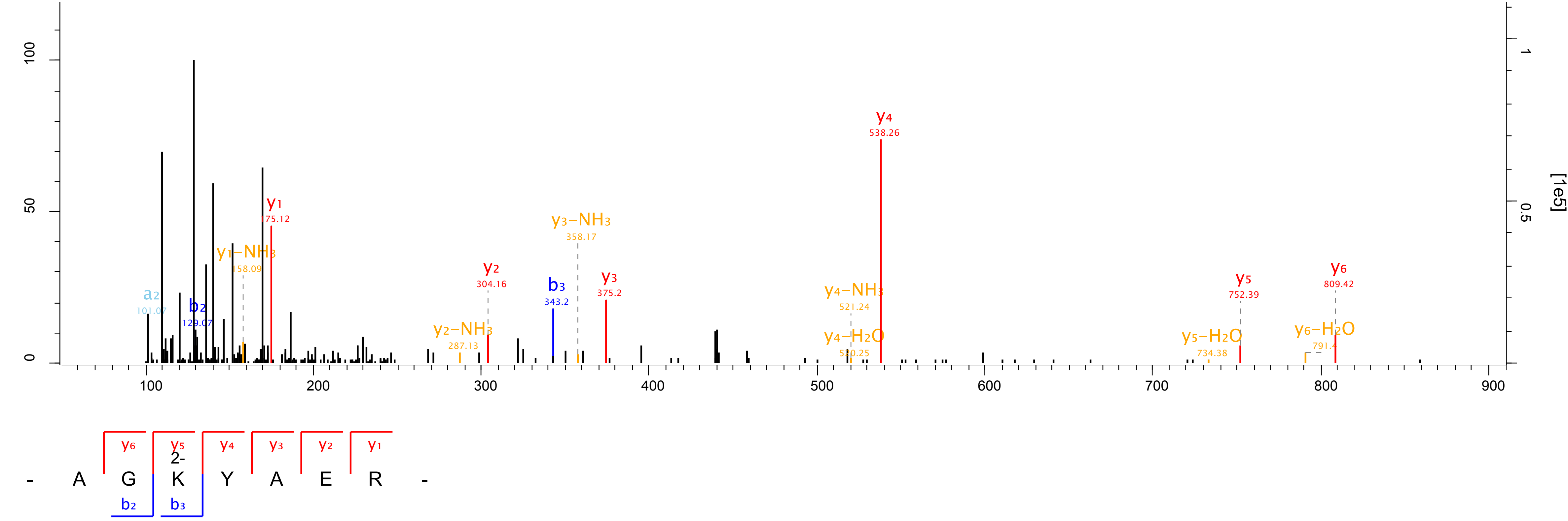

| Raw file | Scan | Method | Score | m/z |
|---|---|---|---|---|
| 3 | 8562 | FTMS; HCD | 67.88 | 658.87 |

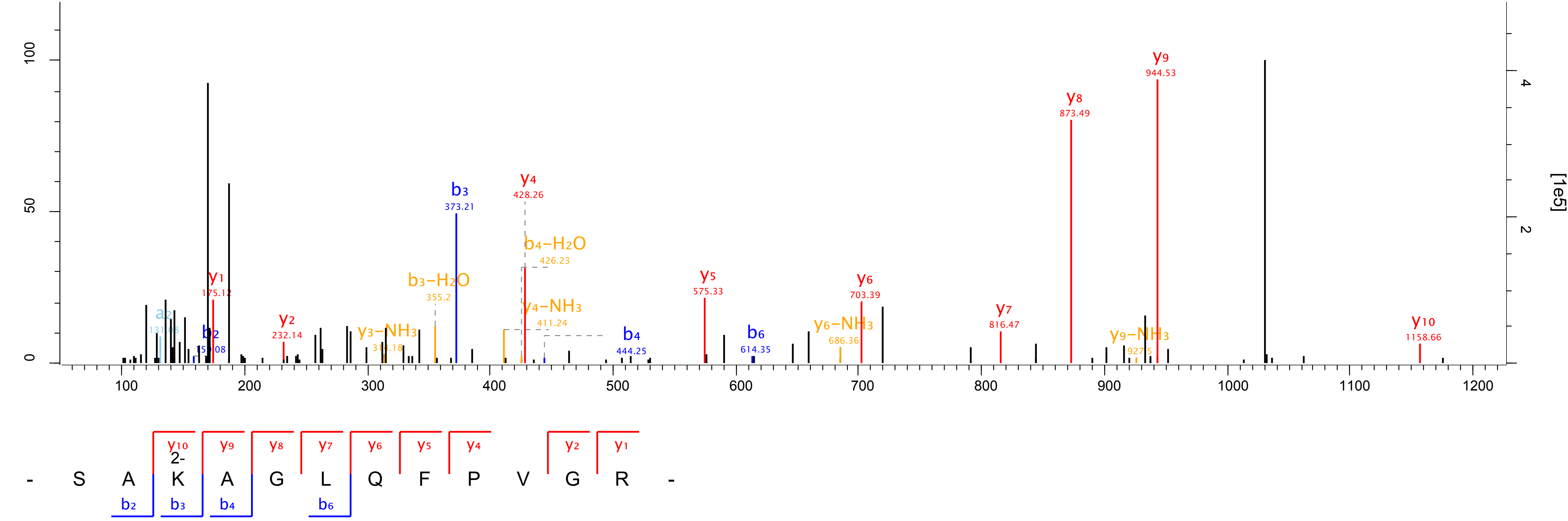

| Raw file | Scan | Method | Score | m/z |
|---|---|---|---|---|
| 4 | 1136 | FTMS; HCD | 78.67 | 486.52 |

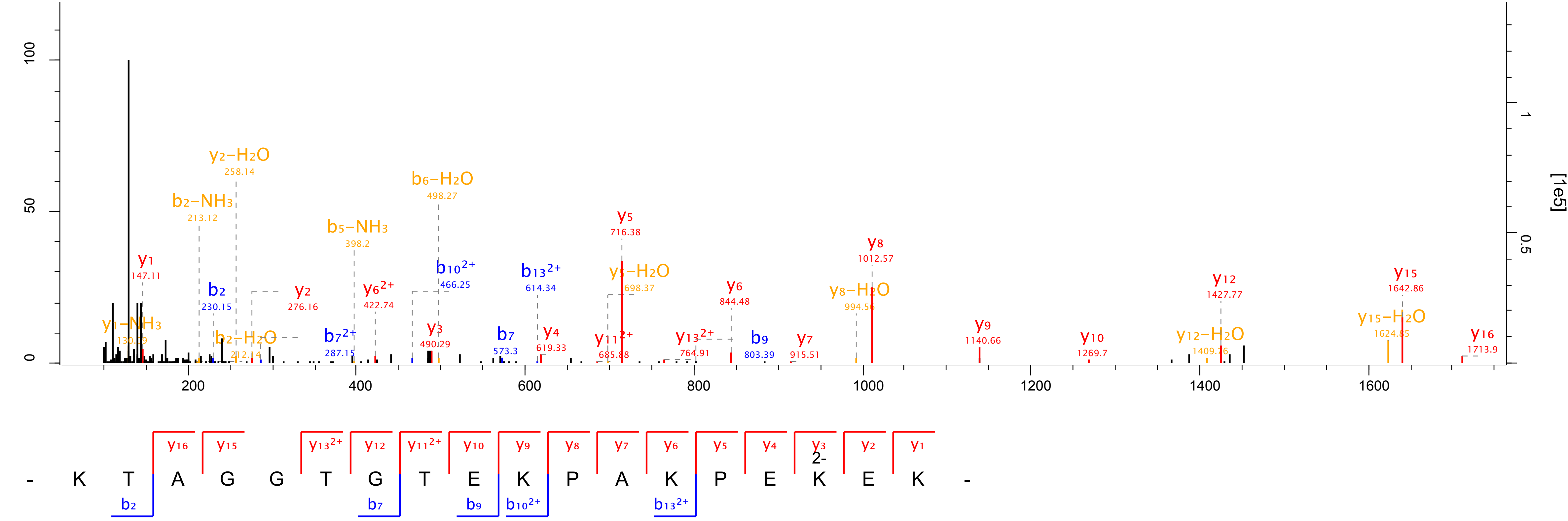

| Raw file | Scan | Method | Score | m/z |
| --- | --- | --- | --- | --- |
| 1 | 2569 | FTMS; HCD | 105.14 | 470.75 |

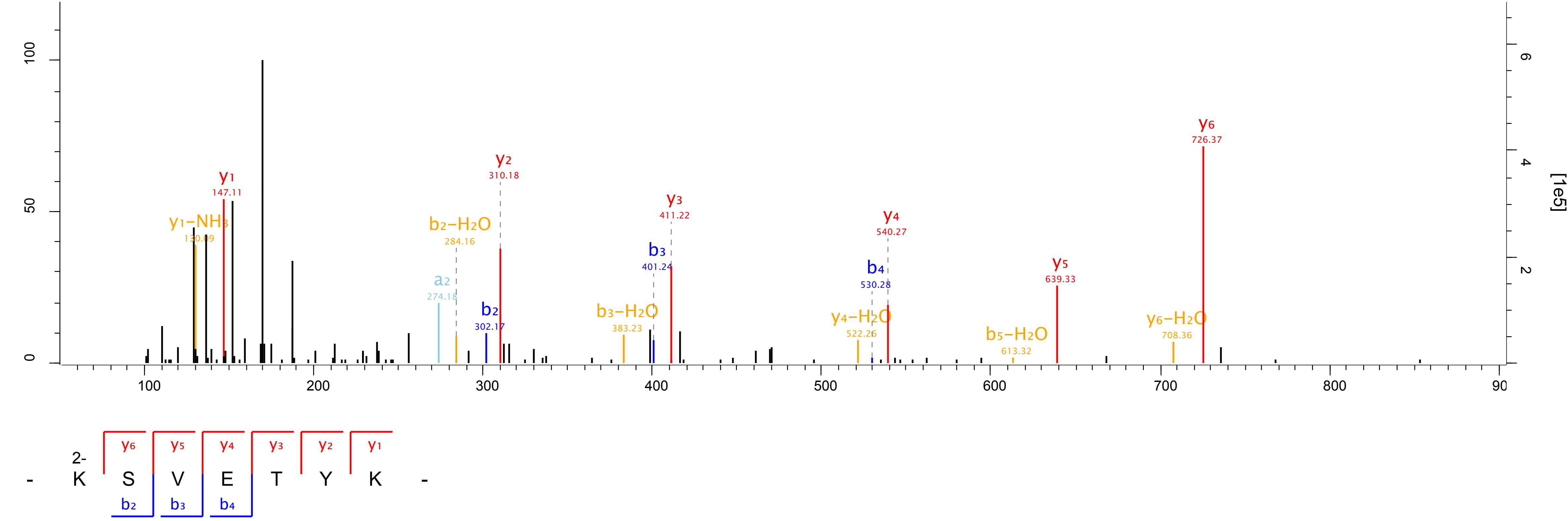

| Raw file | Scan | Method | Score | m/z |
| --- | --- | --- | --- | --- |
| 2 | 7857 | FTMS; HCD | 49.63 | 541.3 |

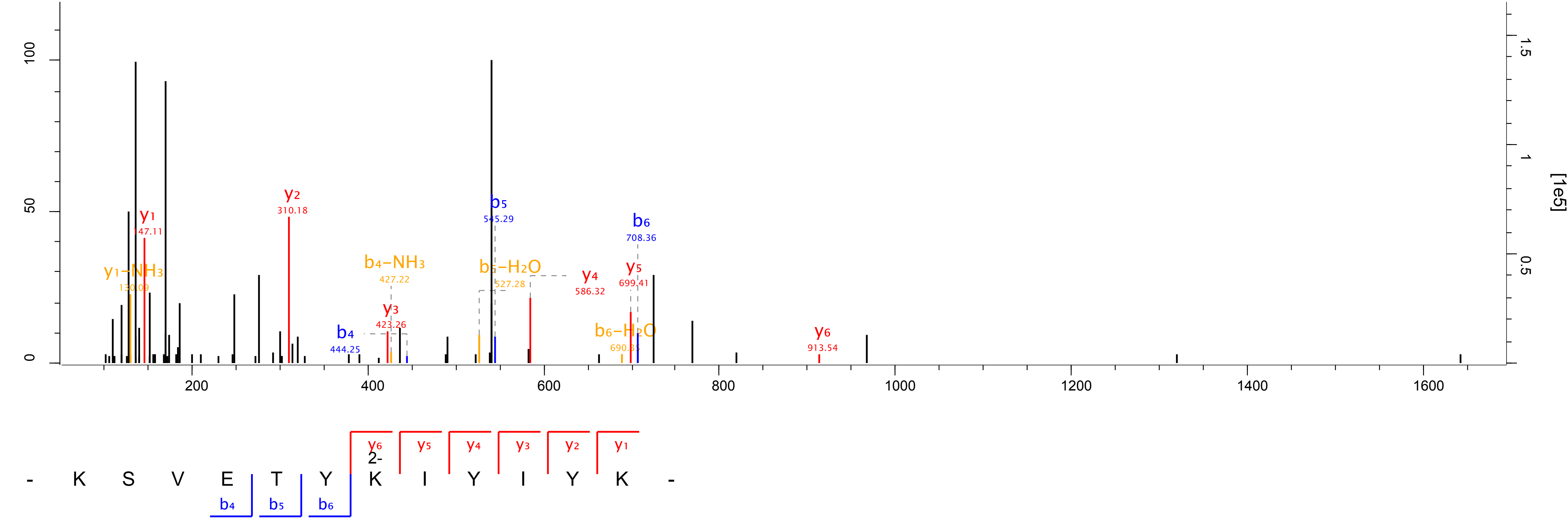

| Raw file | Scan | Method | Score | m/z |
|---|---|---|---|---|
| 6 | 3762 | FTMS; HCD | 198.87 | 797.95 |

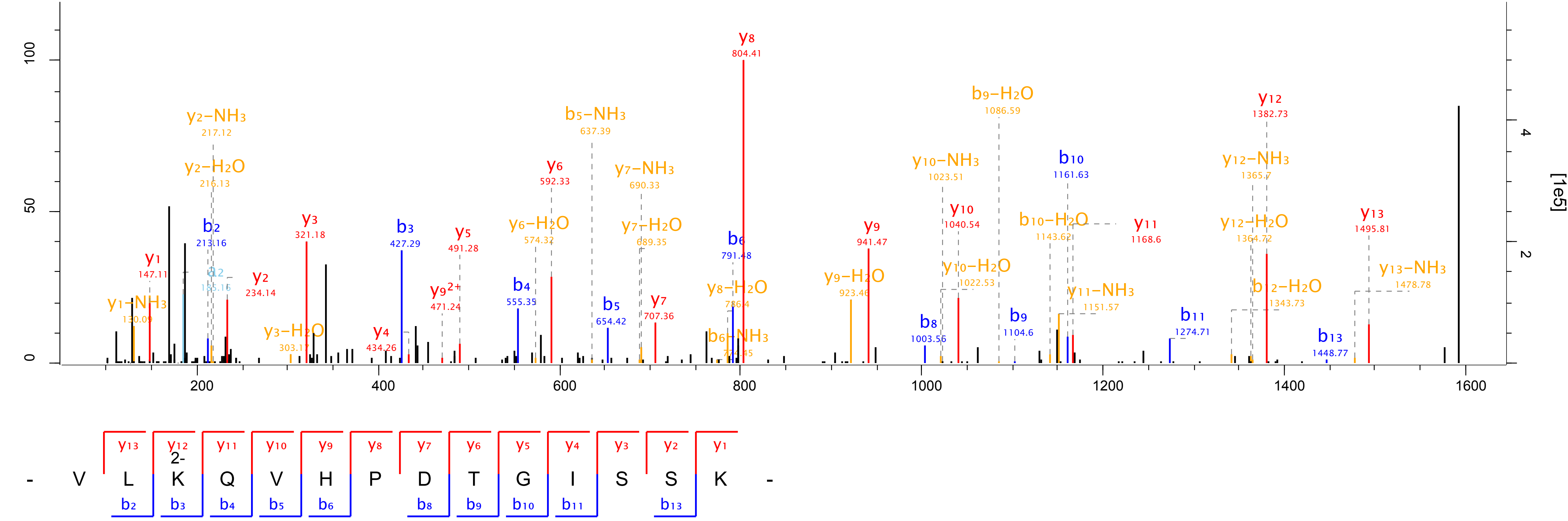

| Raw file | Scan | Method | Score | m/z |
|---|---|---|---|---|
| 2 | 13041 | FTMS; HCD | 87.2 | 1000.16 |

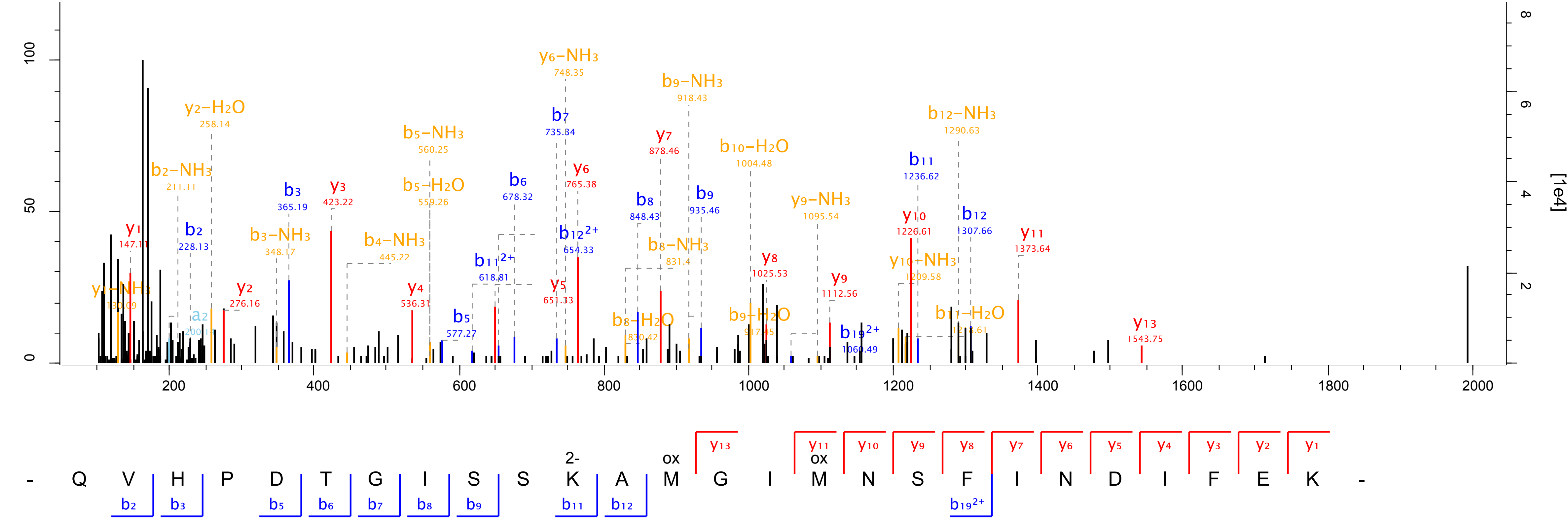

| Raw file | Scan | Method | Score | m/z |
|---|---|---|---|---|
| 2 | 2553 | FTMS; HCD | 104.43 | 647.36 |

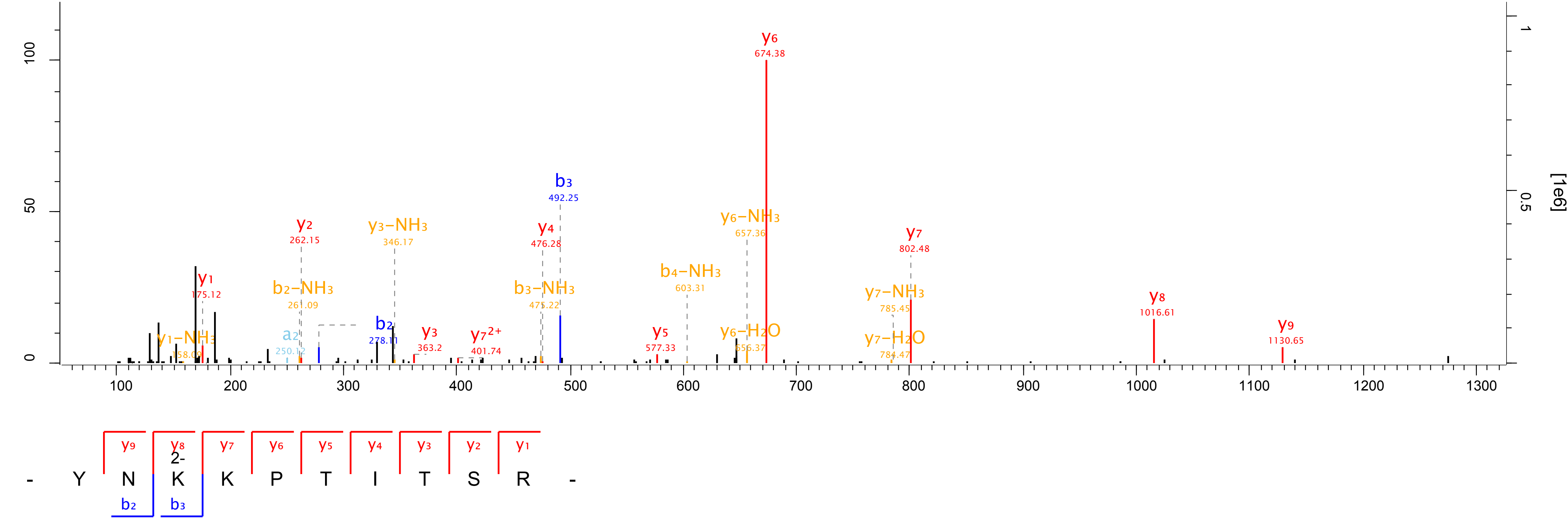

| Raw file | Scan | Method | Score | m/z |
| --- | --- | --- | --- | --- |
| 4 | 9960 | FTMS; HCD | 112.67 | 925.53 |

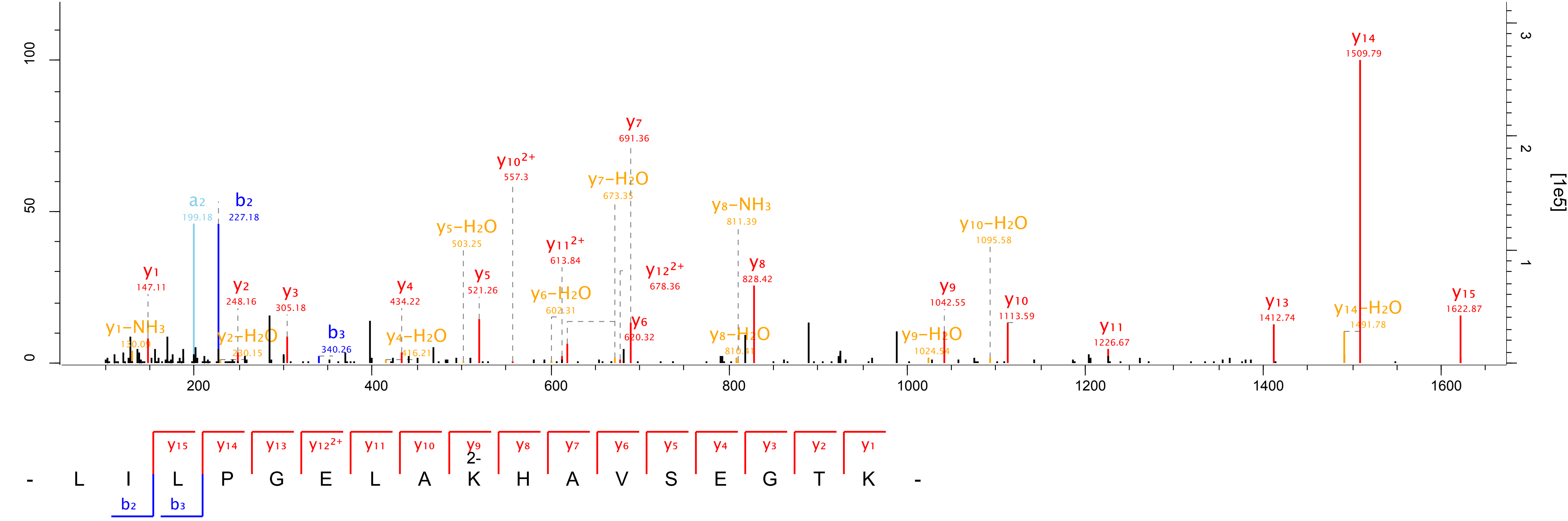

| Raw file | Scan | Method | Score | m/z |
|---|---|---|---|---|
| 1 | 1739 | FTMS; HCD | 205.31 | 657.36 |

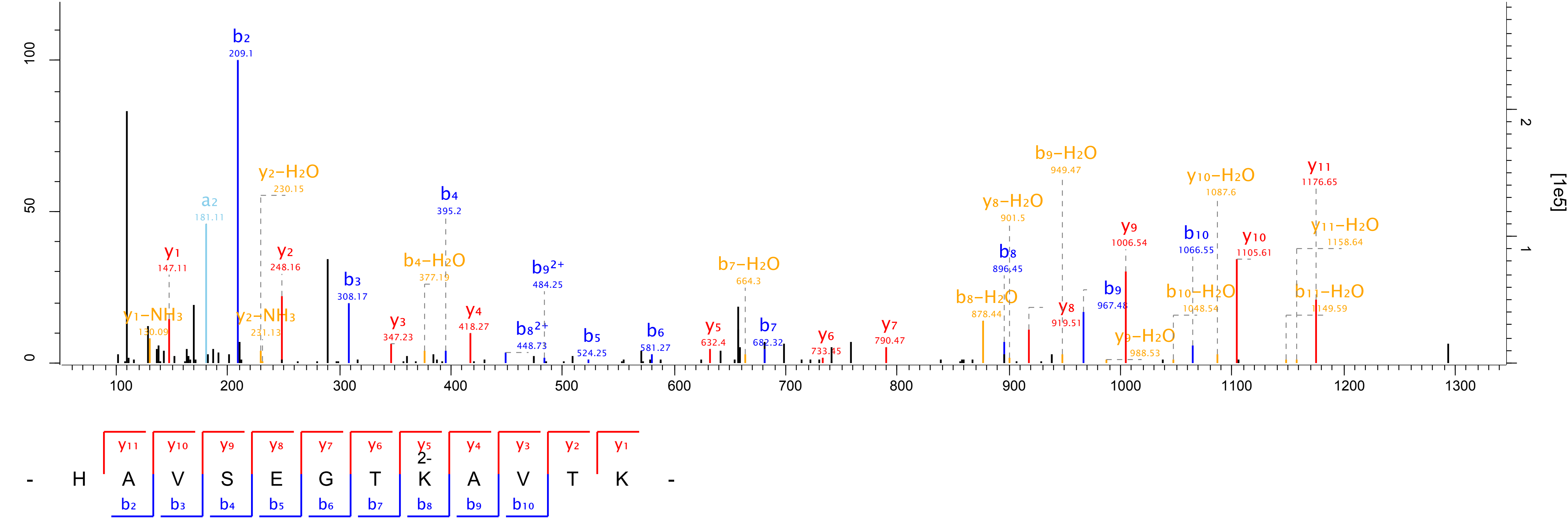

| Raw file | Scan | Method | Score | m/z |
|---|---|---|---|---|
| 6 | 9967 | FTMS; HCD | 103.76 | 770.92 |

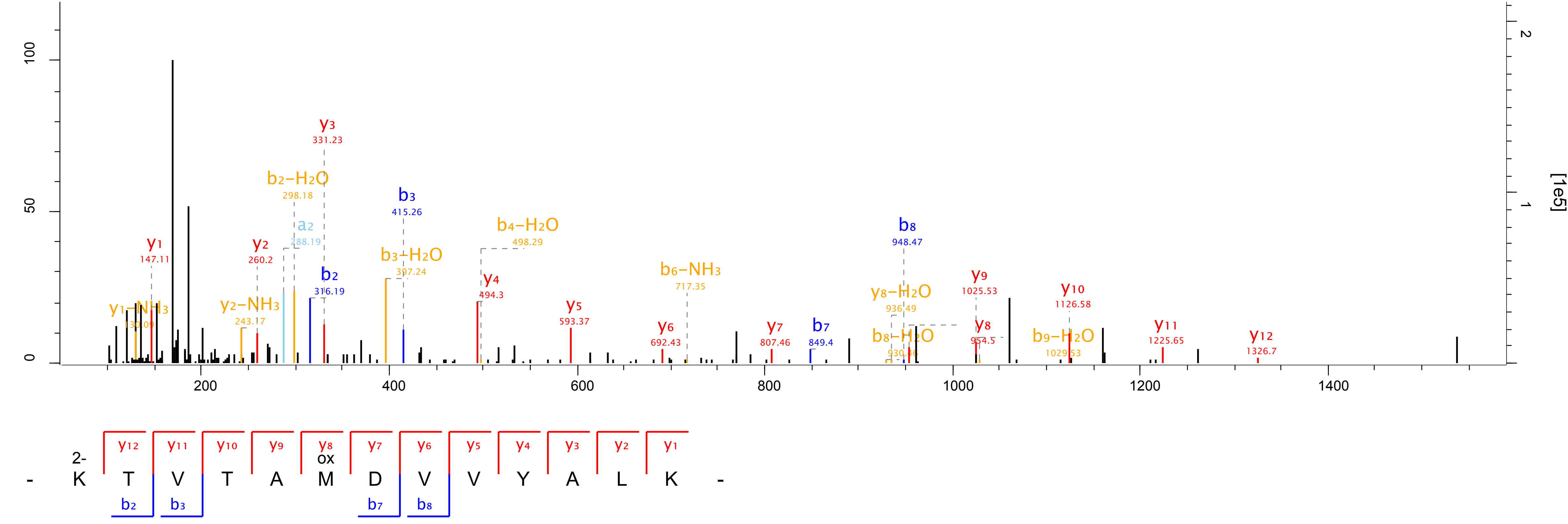

| Raw file | Scan | Method | Score | m/z |
|---|---|---|---|---|
| 3 | 8477 | FTMS; HCD | 117.79 | 706.4 |

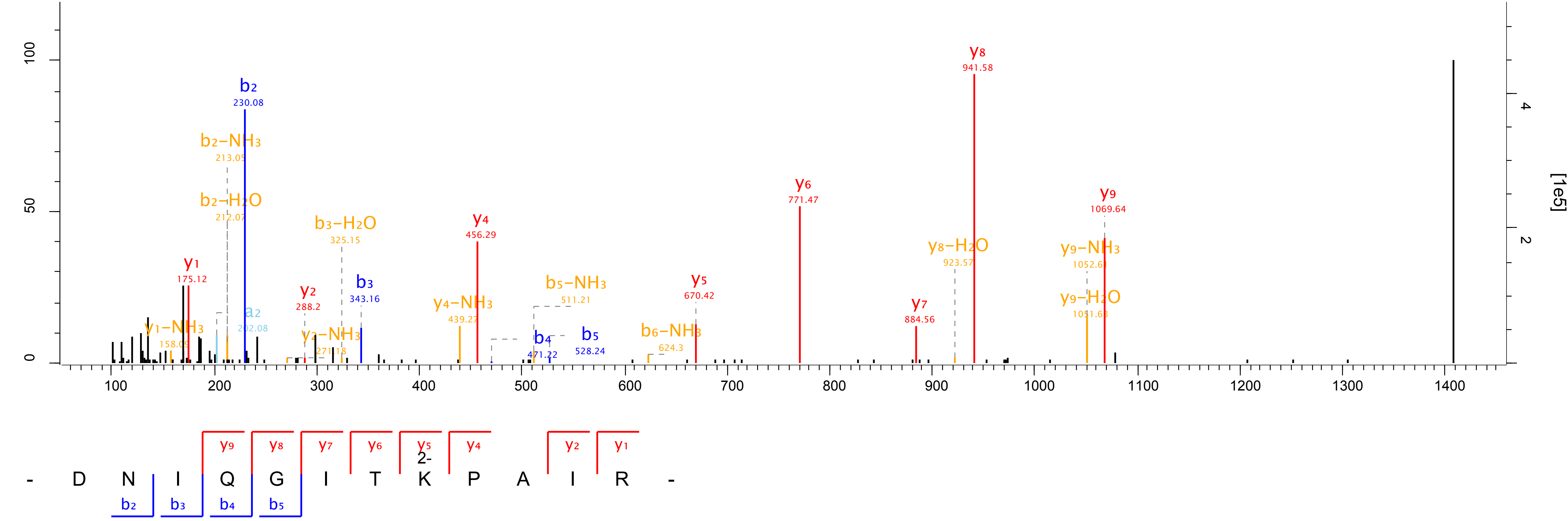

| Raw file | Scan | Method | Score | m/z |
|---|---|---|---|---|
| 6 | 13135 | FTMS; HCD | 138.42 | 776.93 |

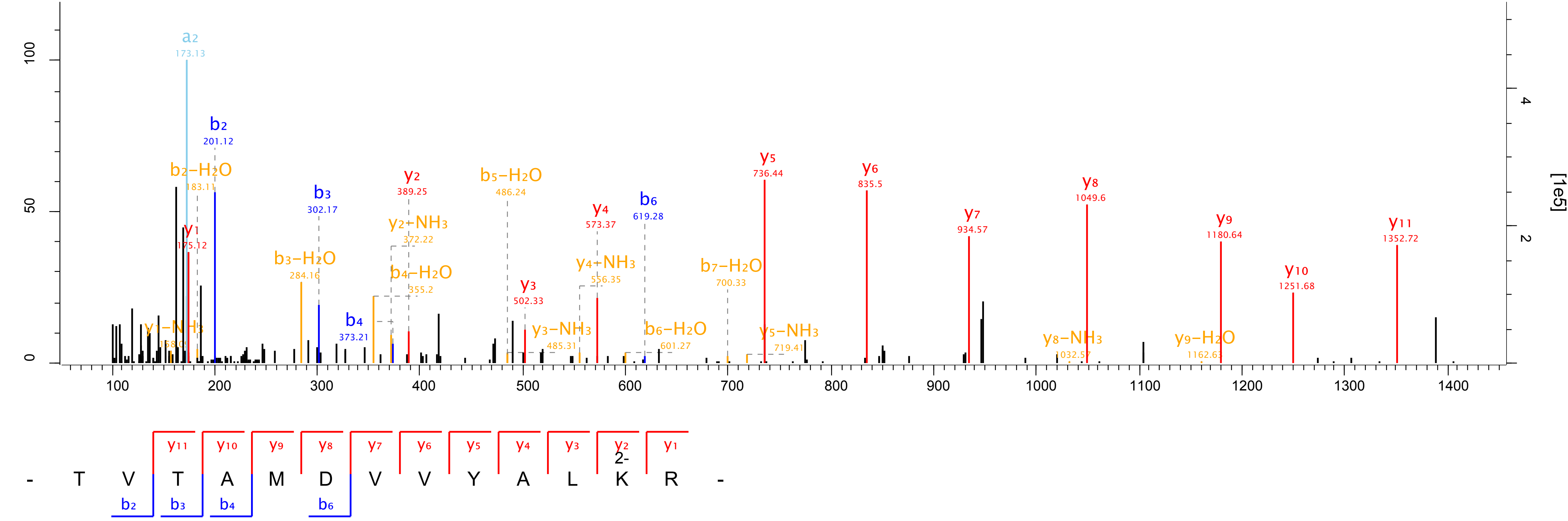

| Raw file | Scan | Method | Score | m/z |
| --- | --- | --- | --- | --- |
| 2 | 12903 | FTMS; HCD | 55.3 | 640.1 |

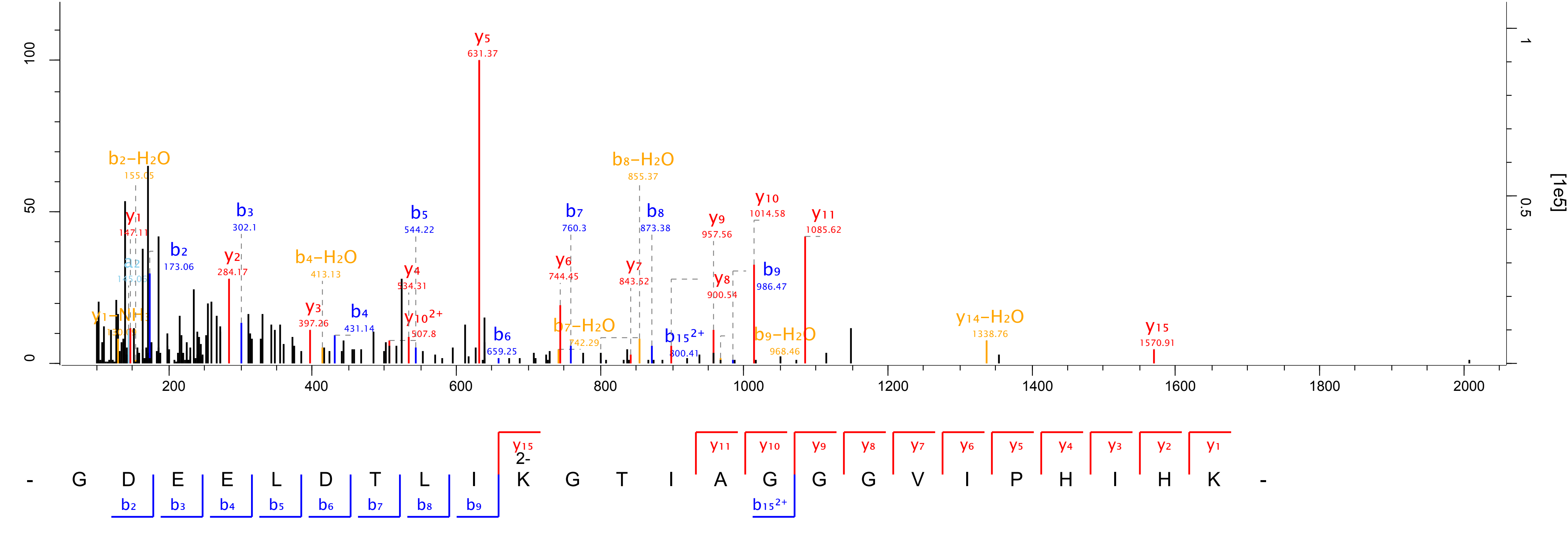

| Raw file | Scan | Method | Score | m/z |
| --- | --- | --- | --- | --- |
| 1 | 8817 | FTMS; HCD | 99.54 | 743.92 |

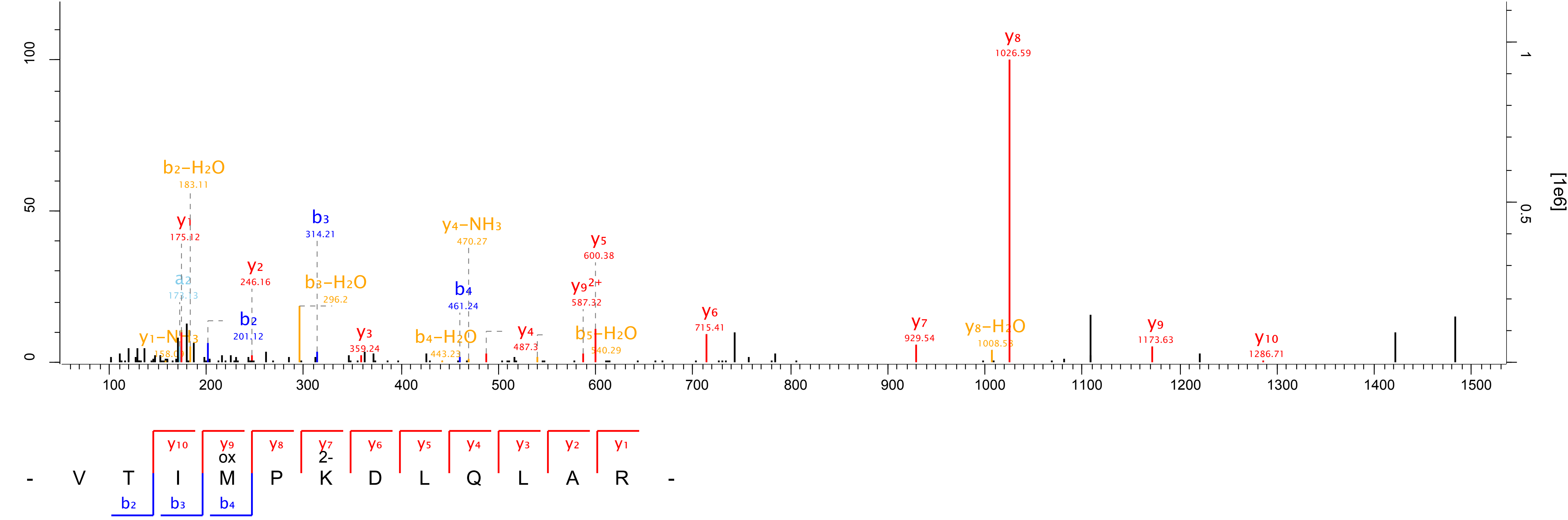

# Figure S2

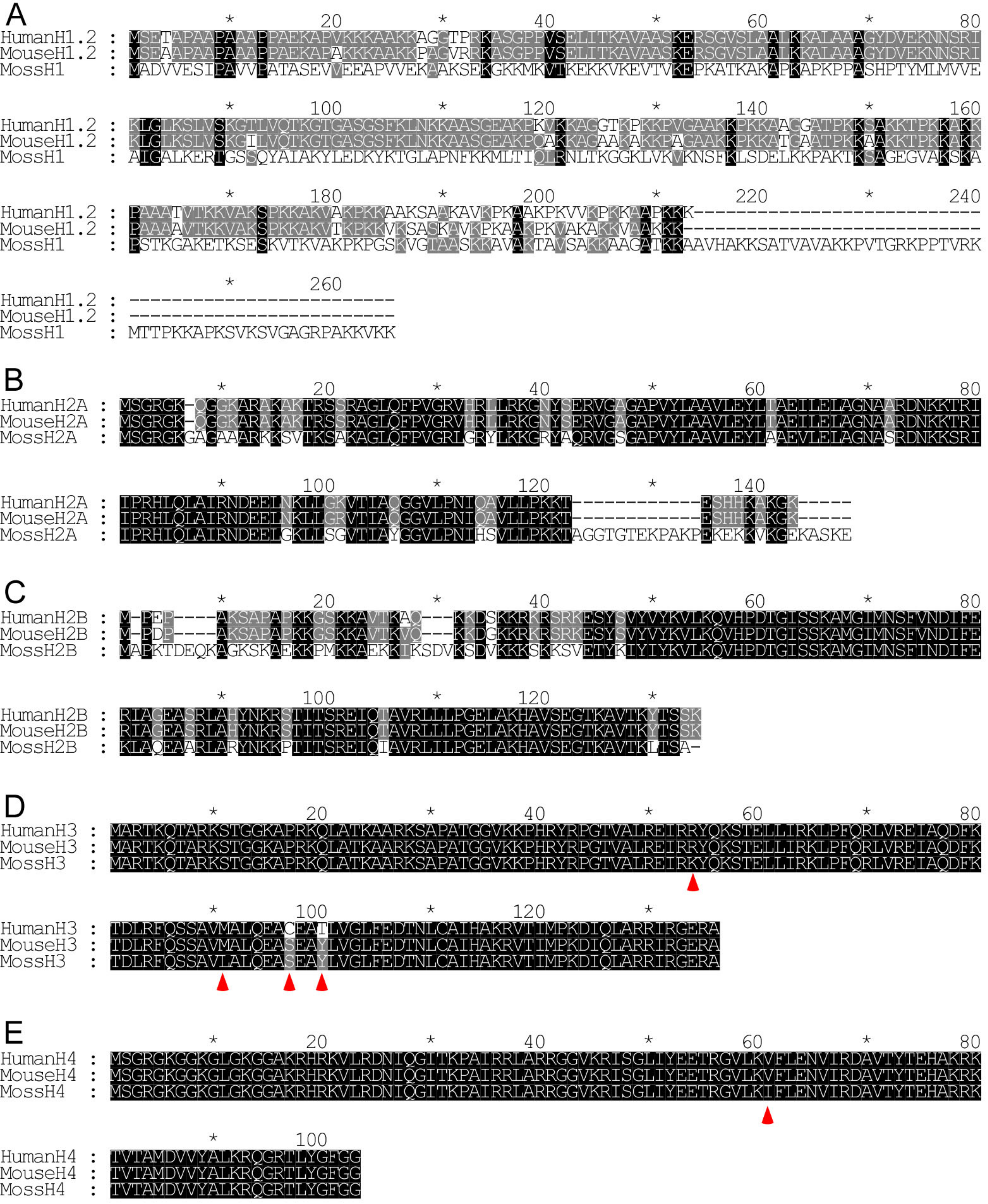
A
HumanH1.2 : MSETAPAAPAAPPAEKAPVKKKAAKKAGGTPRKASGPPVSELITKAVAASKERSGVSLAALKKALAAAGYDVEKNNSRI
MouseH1.2 : MSEAAPAAPAAPPAEKAPAKKKAAKKPAGVRRKASGPPVSELITKAVAASKERSGVSLAALKKALAAAGYDVEKNNSRI
MossH1 : MADVVESIPAVVPATASEVVEEAPVVEKAAKSEKGKKMKVTKEKKVKEVTVKEPKATKAKAPKAPKPPASHPTYMLMVVE
HumanH1.2 : KLGLKSLVSKGTLVQTKGTGASGSFKLNKKAASGEAKPKVKKAGGTKPKKPVGAAKKPKKAAGGATPKKSAKKTPKKAKK
MouseH1.2 : KLGLKSLVSKGILVQTKGTGASGSFKLNKKAASGEAKPQAKKAGAAKAKKPAGAAKKPKKATGAATPKKAAKKTPKKAKK
MossH1 : AIGALKERIGSSQYAIAKYLEDKYKTGLAPNFKKMLTIQLRNLTKGGKLVKVKNSFKLSDELKKPAKTKSAGEGVAKSKA
HumanH1.2 : PAAATVTKKVAKSPKKAKVAKPKKAAKSAAKAVKPKAAKPKVVKPKKAAPKKK---------------------------
MouseH1.2 : PAAAAVTKKVAKSPKKAKVTKPKKVKSASKAVKPKAAKPKVAKAKKVAAKKK----------------------------
MossH1 : PSTKGAKETKSESKVTKVAKPKPGSKVGTAASKKAVAKTAVSAKKAAGATKKAAVHAKKSATVAVAKKPVTGRKPPTVRK
HumanH1.2 : -------------------------
MouseH1.2 : -------------------------
MossH1 : MTTPKKAPKSVKSVGAGRPAKKVKK
B
HumanH2A : MSGRGK-QGGKARAKAKTRSSRAGLQFPVGRVHRLLRKGNYSERVGAGAPVYLAAVLEYLTAEILELAGNAARDNKKTRI
MouseH2A : MSGRGK-QGGKARAKAKTRSSRAGLQFPVGRVHRLLRKGNYSERVGAGAPVYLAAVLEYLTAEILELAGNAARDNKKTRI
MossH2A : MSGRGKGAGAAARKKSVTKSAKAGLQFPVGRLGRYLKKGRYAQRVGSGAPVYLAAVLEYLAAEVLELAGNASRDNKKSRI
HumanH2A : IPRHLQLAIRNDEELNKLLGKVTIAQGGVLPNIQAVLLPKKT------------ESHHKAKGK-----
MouseH2A : IPRHLQLAIRNDEELNKLLGRVTIAQGGVLPNIQAVLLPKKT------------ESHHKAKGK-----
MossH2A : IPRHIQLAIRNDEELGKLLSGVTIAYGGVLPNIHSVLLPKKTAGGTGTEKPAKPEKEKKVKGEKASKE
C
HumanH2B : M-PEP----AKSAPAPKKGSKKAVTKAQ---KKDSKKRKRSRKESYSVYVYKVLKQVHPDTGISSKAMGIMNSFVNDIFE
MouseH2B : M-PDP----AKSAPAPKKGSKKAVTKVQ---KKDGKKRKRSRKESYSVYVYKVLKQVHPDTGISSKAMGIMNSFVNDIFE
MossH2B : MAPKTDEQKAGKSKAEKKPMKKAEKKIKSDVKSDVKKKSKKSVETYKIYIYKVLKQVHPDTGISSKAMGIMNSFINDIFE
HumanH2B : RIAGEASRLAHYNKRSTITSREIQTAVRLLLPGELAKHAVSEGTKAVTKYTSSK
MouseH2B : RIAGEASRLAHYNKRSTITSREIQTAVRLLLPGELAKHAVSEGTKAVTKYTSSK
MossH2B : KLAQEAARLARYNKKPTITSREIQIAVRLILPGELAKHAVSEGTKAVTKLTSA-
D
HumanH3 : MARTKQTARKSTGGKAPRKQLATKAARKSAPATGGVKKPHRYRPGTVALREIRRYQKSTELLIRKLPFQRLVREIAQDFK
MouseH3 : MARTKQTARKSTGGKAPRKQLATKAARKSAPATGGVKKPHRYRPGTVALREIRRYQKSTELLIRKLPFQRLVREIAQDFK
MossH3 : MARTKQTARKSTGGKAPRKQLATKAARKSAPATGGVKKPHRYRPGTVALREIRKYQKSTELLIRKLPFQRLVREIAQDFK
HumanH3 : TDLRFQSSAVMALQEACEATLVGLFEDTNLCAIHAKRVTIMPKDIQLARRIRGERA
MouseH3 : TDLRFQSSAVMALQEASEAYLVGLFEDTNLCAIHAKRVTIMPKDIQLARRIRGERA
MossH3 : TDLRFQSSAVLALQEASEAYLVGLFEDTNLCAIHAKRVTIMPKDIQLARRIRGERA
E
HumanH4 : MSGRGKGGKGLGKGGAKRHRKVLRDNIQGITKPAIRRLARRGGVKRISGLIYEETRGVLKVFLENVIRDAVTYTEHAKRK
MouseH4 : MSGRGKGGKGLGKGGAKRHRKVLRDNIQGITKPAIRRLARRGGVKRISGLIYEETRGVLKVFLENVIRDAVTYTEHAKRK
MossH4 : MSGRGKGGKGLGKGGAKRHRKVLRDNIQGITKPAIRRLARRGGVKRISGLIYEETRGVLKIFLENVIRDAVTYTEHARRK
HumanH4 : TVTAMDVVYALKRQGRTLYGFGG
MouseH4 : TVTAMDVVYALKRQGRTLYGFGG
MossH4 : TVTAMDVVYALKRQGRTLYGFGG

# Figure S3

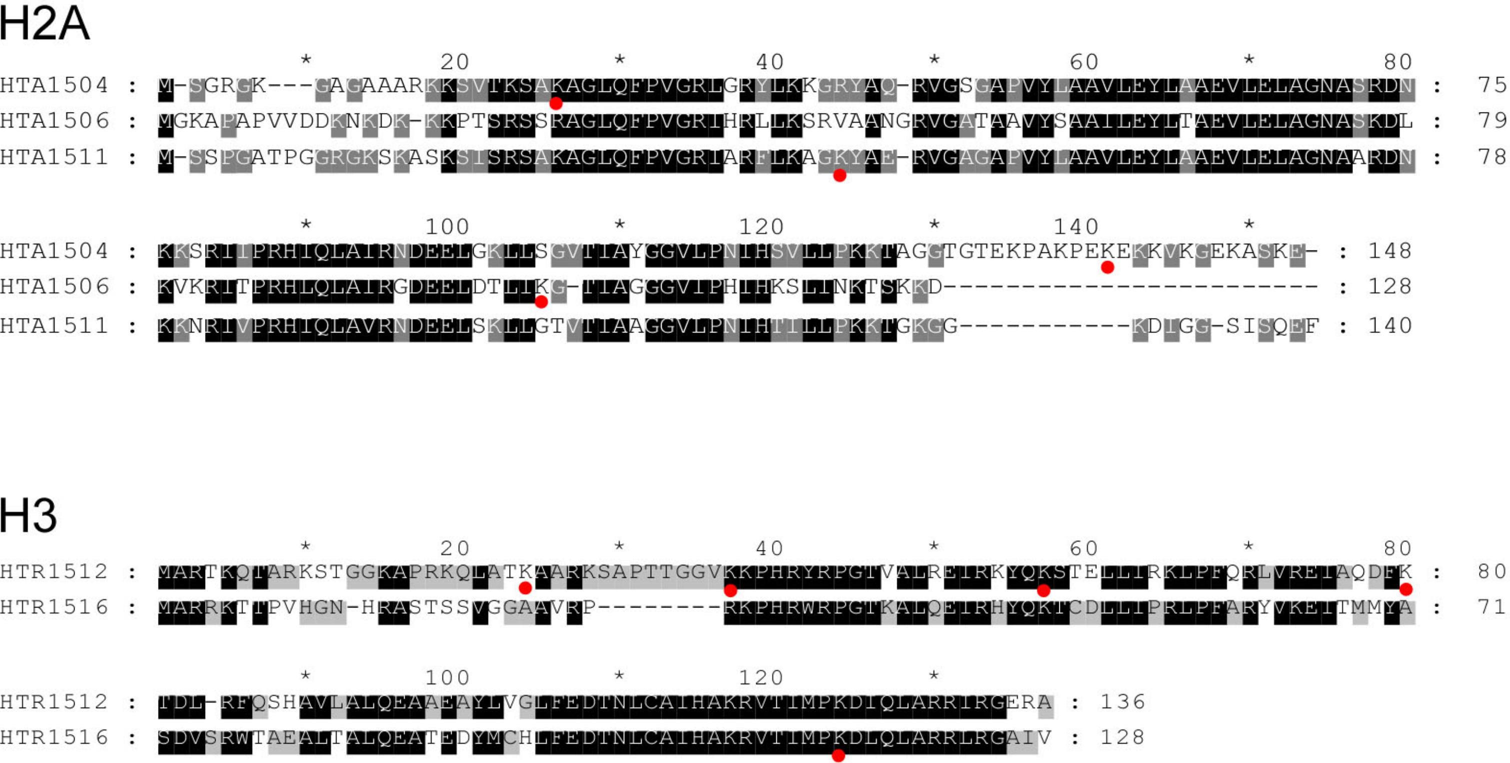
H2A
* 20 * 40 * 60 * 80
HTA1504 : M-SGRGK---GAGAAARKKSVTKSAKAGLQFPVGRLGRYLKKGRYAQ-RVGSGAPVYLAAVLEYLAAEVLELAGNASRDN : 75
HTA1506 : MGKAPAPVVDDKNKDK-KKPTSRSSRAGLQFPVGRIHRLLKSRVAANGRVGATAAVYSAAILEYLTAEVLELAGNASKDL : 79
HTA1511 : M-SSPGATPGGRGKSKASKSTSRSAKAGLQFPVGRIARFLKAGKYAE-RVGAGAPVYLAAVLEYLAAEVLELAGNAARDN : 78
* 100 * 120 * 140 *
HTA1504 : KKSRIIPRHIQLAIRNDEELGKLLSGVTIAYGGVLPNIHSVLLPKKTAGGTGTEKPAKPEKEKKVKGEKASKE- : 148
HTA1506 : KVKRITPRHLQLAIRGDEELDTLIKG-TIAGGGVIPHIHKSLINKTSKKD------------------------ : 128
HTA1511 : KKNRIVPRHIQLAVRNDEELSKLLGTVTIAAGGVLPNIHTILLPKKTGKGG-----------KDIGG-SISQEF : 140
H3
* 20 * 40 * 60 * 80
HTR1512 : MARTKQTARKSTGGKAPRKQLATKAARKSAPTTGGVKKPHRYRPGTVALREIRKYQKSTELLIRKLPFQRLVREIAQDFK : 80
HTR1516 : MARRKTTPVHGN-HRASTSSVGGAAVRP--------RKPHRWRPGTKALQEIRHYQKTCDLLIPRLPFARYVKEITMMYA : 71
* 100 * 120 *
HTR1512 : TDL-RFQSHAVLALQEAAEAYLVGLFEDTNLCAIHAKRVTIMPKDIQLARRIRGERA : 136
HTR1516 : SDVSRWTAEALTALQEATEDYMCHLFEDTNLCAIHAKRVTIMPKDLQLARRLRGAIV : 128

# Figure S4

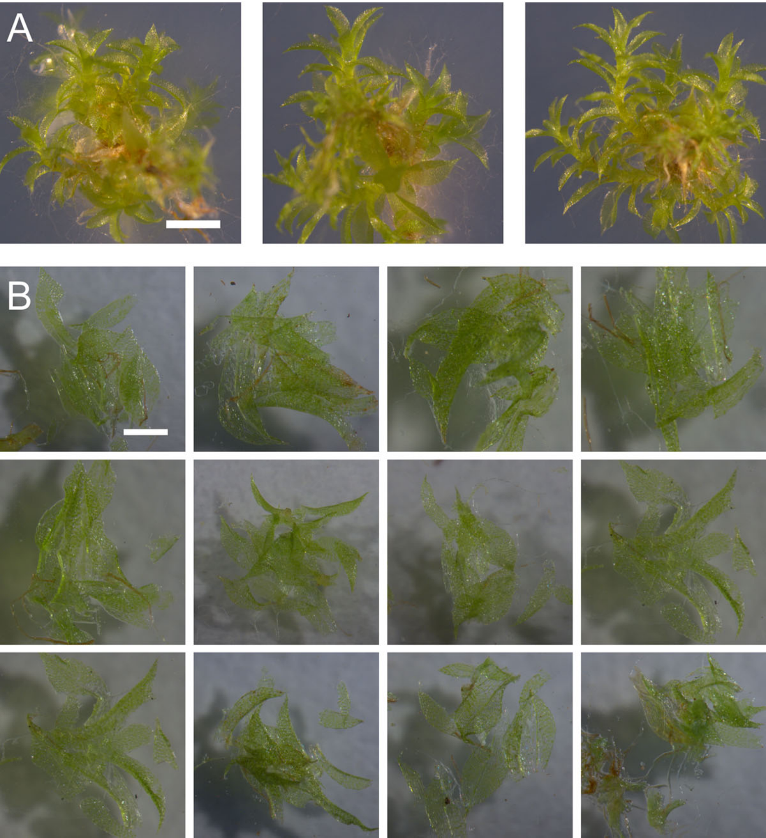

Supplement: Supplementary file 1 — Supplementary figures [file 41598_2017_15854_MOESM1_ESM.pdf]
